# PlantGPT: An Arabidopsis‐Based Intelligent Agent that Answers Questions about Plant Functional Genomics

**DOI:** 10.1002/advs.202503926

**Published:** 2025-05-21

**Authors:** Ruixiang Zhang, Yu Wang, Weiyang Yang, Jun Wen, Weizhi Liu, Shipeng Zhi, Guangzhou Li, Nan Chai, Jiaqi Huang, Yongyao Xie, Xianrong Xie, Letian Chen, Miao Gu, Yao‐Guang Liu, Qinlong Zhu

**Affiliations:** ^1^ Guangdong Basic Research Center of Excellence for Precise Breeding of Future Crops Guangdong Laboratory for Lingnan Modern Agriculture State Key Laboratory for Conservation and Utilization of Subtropical Agro‐Bioresources College of Agriculture College of Life Science South China Agricultural University Guangzhou 510642 China; ^2^ School of Life Sciences Institute for Immunology State Key Laboratory of Membrane Biology China Ministry of Education Key Laboratory of Protein Sciences Tsinghua University Beijing 100084 China; ^3^ Department of Automation Tsinghua University Beijing 100084 China; ^4^ Department of Medicine Tsinghua University Beijing 100084 China; ^5^ Engineering Research Center of Protection and Utilization of Plant Resources College of Bioscience and Biotechnology Shenyang Agricultural University Shenyang 110866 China

**Keywords:** functional genomics, large language models, plant gene function retrieval‐augmented generation

## Abstract

Research into plant gene function is crucial for developing strategies to increase crop yields. The recent introduction of large language models (LLMs) offers a means to aggregate large amounts of data into a queryable format, but the output can contain inaccurate or false claims known as hallucinations. To minimize such hallucinations and produce high‐quality knowledge‐based outputs, the abstracts of over 60 000 plant research articles are compiled into a Chroma database for retrieval‐augmented generation (RAG). Then linguistic data are used from 13 993 Arabidopsis (*Arabidopsis thaliana*) phenotypes and 23 323 gene functions to fine‐tune the LLM Llama3‐8B, producing PlantGPT, a virtual expert in Arabidopsis phenotype–gene research. By evaluating answers to test questions, it is demonstrated that PlantGPT outperforms general LLMs in answering specialized questions. The findings provide a blueprint for functional genomics research in food crops and demonstrate the potential for developing LLMs for plant research modalities. To provide broader access and facilitate adoption, the online tool http://www.plantgpt.icu is developed, which will allow researchers to use PlantGPT in their scientific investigations.

## Graphical abstract

PlantGPT integrates 60 000+ plant research articles with Arabidopsis phenotype‐gene data through retrieval‐augmented generation and fine‐tuning of Llama3‐8B. This open‐source, specialized AI system outperforms general large language models in plant gene‐phenotype relationships, establishing a new paradigm for functional genomics research and molecular design breeding.

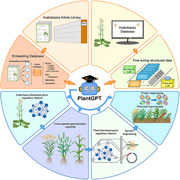

## 1 Introduction

Food crops provide 80% of human dietary needs.^[^ ^1^ ^]^ Modern molecular biology research on functional genomics has made significant contributions to food production by increasing crop yields, nutrient contents, pest resistance, and stress tolerance.^[^ ^2^ ^]^ However, most existing computational biology methods, such as differential expression analysis and pathway enrichment analysis, were originally developed to analyze single‐type omics data independently,^[^ ^3^, ^4^, ^5^ ^]^ making it challenging to analyze and predict the complex regulatory behaviors of biological systems and integrated data obtained using multiomics approaches.^[^ ^6^, ^7^ ^]^ In fact, the field of computational biology faces significant challenges in quantifying the exact contributions of individual factors to gene expression due to multilayered genetic interactions, complex regulatory networks, and tissue‐specific and time‐dependent gene functions.^[^ ^8^, ^9^, ^10^ ^]^ Similarly, complex user interfaces with limited real‐time interactions have restricted the application of traditional computational biology in functional genomics research.^[^ ^11^ ^]^ Furthermore, current bioinformatics tools do not efficiently and effectively integrate multiomics data, such that valuable research articles often remain underutilized due to the insufficient processing of published corpora (text for use as training data) in plant sciences.

Research into gene function in the model plant Arabidopsis (*Arabidopsis thaliana*) has largely guided functional studies of almost all monocot and dicot food and horticultural crops, contributing to the discovery and investigation of genes related to key agronomic traits such as growth and development, yield, and biotic and abiotic stress resistance in crops.^[^ ^12^, ^13^ ^]^ However, traditional databases, such as The Arabidopsis Information Resource (TAIR)^[^ ^14^ ^]^ and the RIKEN Arabidopsis Genome Encyclopedia (RARGE)^[^ ^15^ ^]^ store data in precisely packaged forms but with relatively low interactivity. For example, researchers typically need to know the exact trait name or the precise gene name and gene ID in order to perform a search on the website. This type of limited interface restricts interactivity and may even prevent researchers new to a particular species from obtaining effective information.

Large language models (LLMs) have rapidly developed across various fields,^[^ ^16^, ^17^, ^18^ ^]^ leading to significant advancements in natural language processing, medical diagnosis, autonomous driving, and biological research.^[^ ^19^, ^20^, ^21^, ^22^ ^]^ For instance, CRISPR‐GPT was developed to help researchers quickly navigate and select suitable gene‐editing systems.^[^ ^23^ ^]^ Similarly, the multifunctional LLM ChemLLM was developed based on specialized chemical knowledge.^[^ ^24^ ^]^ BioGPT, an LLM trained on 15 million biomedical‐related articles from PubMed, demonstrated good ability to answer professional knowledge questions,^[^ ^25^ ^]^ which prompted our desire to train specialized LLMs. Another LLM‐based database, BiomedGPT, uses LLMs to identify images and analyze them to generate a medical diagnosis, with accuracy nearly equivalent to that of human experts.^[^ ^21^ ^]^

Several challenges persist in developing LLMs for plant biology studies. For example, limited training data are available based on information about plant research, and insufficient attention has focused on the application of LLMs to agriculture compared to fields, such as chemistry, medicine, and molecular biology.^[^ ^22^, ^26^ ^]^ Moreover, the exploration of the applicability of LLMs to agricultural research has been limited,^[^ ^26^ ^]^ and these tools have shown limited effectiveness in specialized areas requiring high accuracy.^[^ ^27^ ^]^ Finally, incorrect or made‐up information (collectively referred to as hallucinations) caused by both the misunderstanding of domain‐specific terminology and inherent model characteristics remains an important issue.^[^ ^28^ ^]^

**Figure 1:**
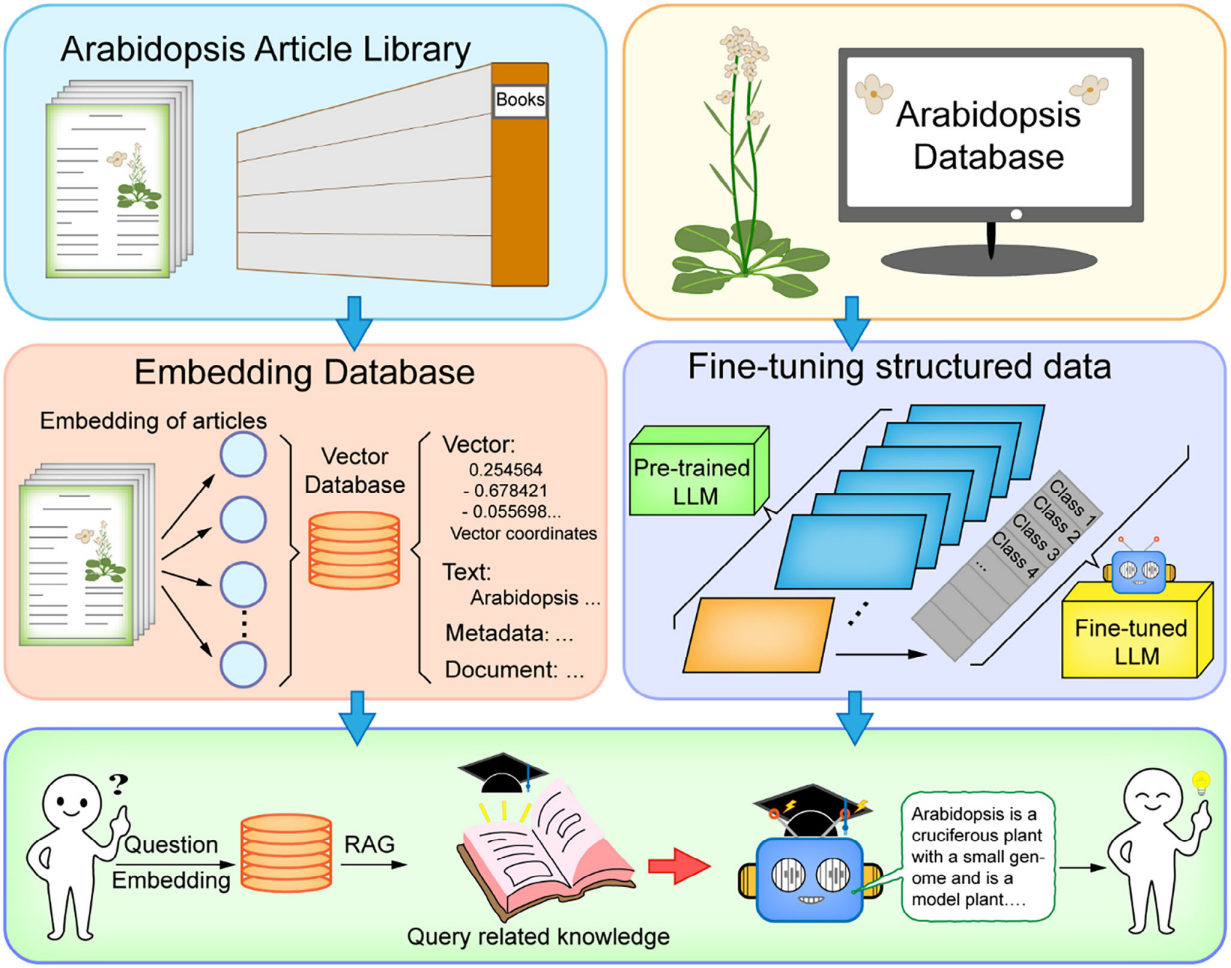
Technical workflow of PlantGPT. LLM, large language model; RAG, retrieval‐augmented generation.

To address these challenges and promote accessibility to LLMs for fostering progress in plant science research, we developed PlantGPT, a virtual expert in Arabidopsis phenotype–gene research, by establishing a vector database (a specialized database that converts text into numerical representations called vectors for efficient similarity searches) of abstracts from numerous published articles and fine‐tuning the LLM Llama3 8B using gene research corpora (**Figure** **1**; and Table S1, Supporting Information). Our work enhances the interaction level between users and plant databases through LLM dialog downstream tasks. Through fine‐tuning and retrieval‐augmented generation (RAG, a technique that retrieves context from our vector database to enhance response accuracy), we achieve real‐time updates and integration of the plant science literature, converting complex data into comprehensive summaries with direct links to articles. This approach effectively addresses complex reasoning problems in the plant science field while limiting hallucinations, bridging the gap between LLMs and plant functional genomics research. Through a two‐stage fine‐tuning approach using both phenotype–gene data and gene functional annotations, our dual approach (RAG5 + fine‐tuned Llama3) significantly outperforms both standalone RAG with larger models (RAG5 + Opus) and fine‐tuning alone (Llama3‐Arab). We also evaluated the auxiliary effect of specialized domain RAG on general LLMs, tested our model by grading answers to 110 specialized questions and 10 inferential questions, and established evaluation criteria for the answers given to plant‐specialized knowledge questions by LLMs. To facilitate broader access and promote continuous improvement, PlantGPT currently provides a free online service at http://www.plantgpt.icu, which can be viewed without an account, to encourage researchers to leverage this tool in their scientific endeavors.

## 2 Results

### 2.1 Optimizing the Construction of a Vector Database for Plant Genomics Literature

As the first step in our approach, we established a high‐quality knowledge base from the available Arabidopsis literature to minimize hallucinations and integrate conclusions from multiple research articles. We identified over 70 000 articles published over the past 30 years (1992–2024) by searching the PubMed database. To ensure high data quality and to allow for efficient data retrieval, we implemented a systematic journal selection criterion based on the Journal Citation Reports (JCR) rankings published by Clarivate Analytics (June 2024). Specifically, we selected journals ranked within the Q1–Q3 quartiles and the top 60% of journals in the plant sciences (Table S1, Supporting Information). We also separately evaluated multidisciplinary journals based on their contributions to Arabidopsis research. This analysis yielded 60 429 high‐quality articles from authoritative journals as the core of our knowledge base.

In the second step, we systematically explored the granularity of data contained in the abstract corpus at different levels of segmentation. We tested seven segmentation strategies using 2000 randomly selected abstracts: complete sentence segmentation, fixed token lengths (64 or 128 tokens), and overlapping strategies (30 words with 10‐word overlap [30–10], 50 words with 20‐word overlap [50–20], 50 words with 30‐word overlap [50–30], and 60 words with 40‐word overlap [60–40]) (Table S2, Supporting Information). We evaluated each strategy using a comprehensive scoring system (Table S3, Supporting Information) that assessed scientific accuracy, information coverage, logical coherence, context preservation, and response efficiency. Through rigorous comparisons across 10 questions used for evaluation, we determined that the 50–30 strategy demonstrated superior performance, achieving the highest average score (80.9 ± 5.3, mean ± standard deviation [SD]) out of a possible 100 across all dimensions (Figure S3, Supporting Information). Compared to sentence‐based segmentation (78.7 ± 3.4, mean ± SD), which led to fragmented information retrieval, or longer segments (60–40: 77.8 ± 5.2, mean ± SD), which introduced redundancy, the overlapping strategy of 50 words with 30‐word overlap achieved the optimal balance between content coverage and context preservation. This strategy substantially outperformed the others in maintaining semantic coherence (context preservation: 18.2/20) and information completeness (information coverage: 22.5/25).

In the third step, we constructed a vector database using OpenAI's vector embedding technology to embed the abstracts of these articles after segmentation using the 50–30 strategy, yielding abstract fragments of ≈50 words and a 30‐word overlap between adjacent segments. This collection of 50‐word fragments formed our Arabidopsis knowledge vector database, which we used for RAG based on user queries (Figure 1). We implement a 3‐month (quarterly) update cycle to systematically add newly published articles to the database, ensuring its continued relevance and comprehensiveness.

### 2.2 Evaluating the Effects of RAG Enhancement Across Different LLMs

We developed a retrieval‐augmented language model assistant for plant functional genomics named PlantGPT. To systematically evaluate its performance against other LLMs, we designed a systematic assessment framework for specialized plant genomics queries. Drawing from recent advances in LLM evaluation methodologies,^[^ ^29^ ^]^ we established a 100‐point scoring system across 10 specific dimensions: scientific accuracy (20 points), relevance (15 points), completeness (10 points), depth (10 points), clarity (10 points), up‐to‐dateness (10 points), references (5 points), practicality (10 points), language (5 points), and recognition of limitations (5 points) (Table S4, Supporting Information). This framework was inspired by established evaluation methodologies for domain‐specific LLMs, particularly building upon successful applications in the biomedical field.^[^ ^25^ ^]^

To implement this scoring system, nine domain experts in plant functional genomics—including senior researchers, postdoctoral fellows, and advanced graduate students—independently evaluated 110 specialized knowledge questions, scoring the responses from PlantGPT and each LLM according to detailed rubrics for each scoring dimension. The scientific accuracy dimension primarily evaluates the factual correctness and precision of plant biology knowledge, while the information coverage dimension assesses both the completeness of relevant aspects and the depth of technical details presented in the responses.^[^ ^30^ ^]^

**Figure 2:**
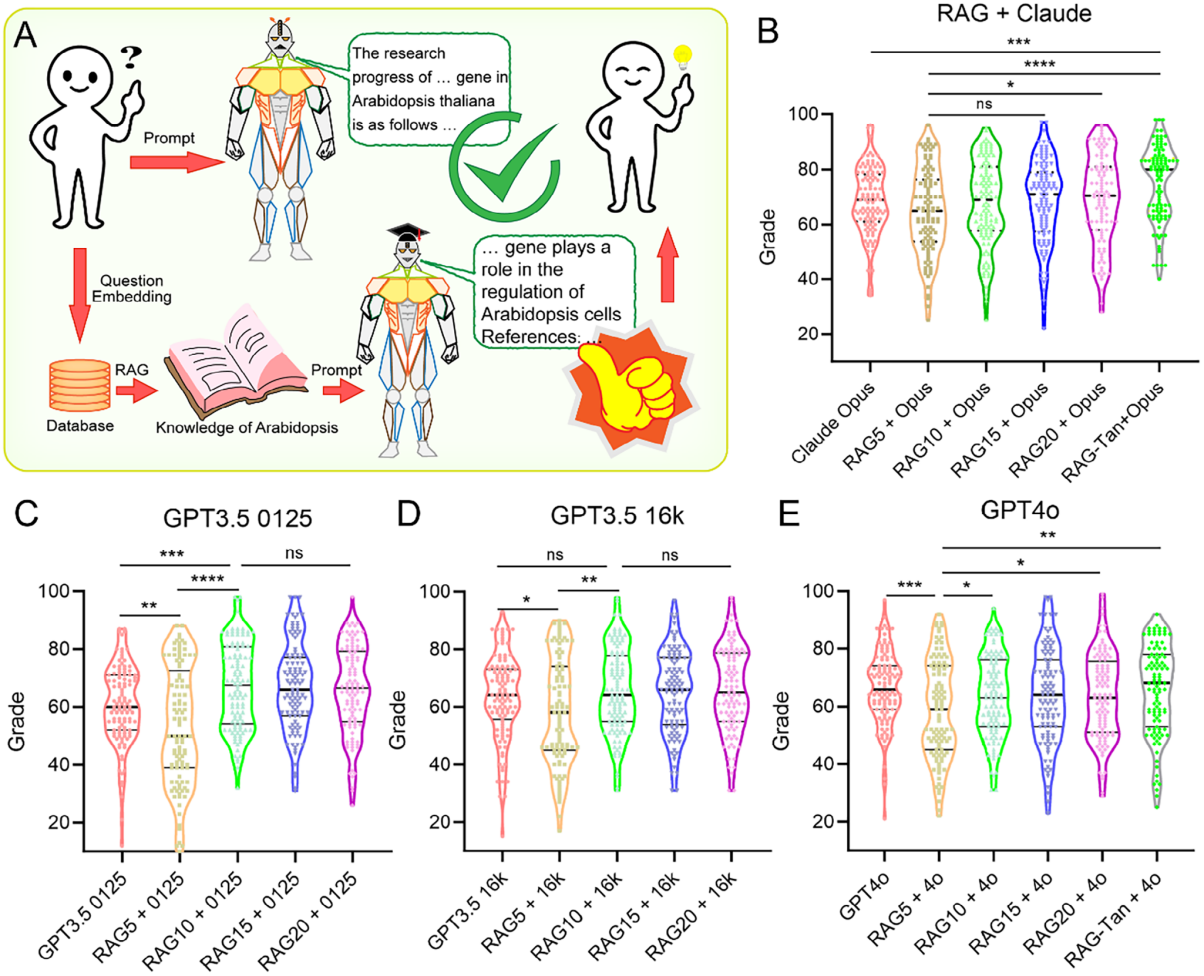
The Arabidopsis vector database enhances the ability of different LLMs to answer specialized questions. A) Pipeline for combining the Arabidopsis knowledge vector database with LLMs to enhance their capability to answer specialized knowledge questions. B–E) Scoring results of the claude‐3‐opus‐20240229 model B), the GPT‐3.5‐turbo‐0125 model C), the GPT‐3.5‐turbo‐16k model D), and the GPT‐4o model E) with RAG in answering Arabidopsis‐related knowledge questions. Data are presented as mean ± SD, *n* = 110. *p*‐values were calculated using unpaired two‐tailed *t*‐test, *p < 0.05, **p < 0.01, ***p < 0.001, ****p < 0.0001. “ns” indicates no significant difference.

Having established this evaluation framework, we conducted a series of experiments to assess whether and how improving RAG recall quality might enhance LLM performance across different models^[^ ^31^ ^]^ (**Figure** **2**A). The granularity of the corpus retrieved by RAG and the amount of input (queries/prompts) affect the quality of LLM responses based on the retrieved knowledge, requiring a dynamic adjustment of input volume based on the task type.^[^ ^32^ ^]^ Therefore, we set up a test with different thresholds of vector retrievals, i.e., retrieving the top five (RAG5), top 10 (RAG10), top 15 (RAG15), top 20 (RAG20), or all vectors before the inflection point of the similarity vector return value (RAG‐Tan). Using our established vector database, we tested the combination of each vector retrieval threshold with Anthropic's claude‐3‐opus‐20240229 model on the 110 specialized questions (Table S5, Supporting Information). Adding the RAG step before the Opus model significantly improved the ability of the model to answer specialized questions about Arabidopsis, with performance increasing proportionally to the RAG vector return values. Notably, the RAG‐Tan + Opus model achieved the best performance (Figure 2B; and Table S1, Supporting Information), demonstrating excellent capability in answering both specialized and general questions (Table S5, Supporting Information).

To further explore the relationship between model capability and RAG effectiveness, we expanded our testing to other OpenAI models: GPT‐3.5‐turbo‐0125, GPT‐3.5‐turbo‐16k, and GPT‐4o. We observed distinct patterns across different model scales. While the combination of each model with RAG5 performed worse than the model alone, in general, increasing the RAG return volume led to improved answer quality for specialized questions (Figure 2; and Table S1, Supporting Information). However, the optimal RAG level varied from one model to another: GPT‐3.5‐turbo‐0125 and GPT‐3.5‐turbo‐16k performed best with moderate RAG input (RAG10 and RAG15, respectively, Figure 2C,D), whereas the GPT‐4o model showed continuous improvement with increasing RAG input, performing best with RAG‐Tan (Figure 2E). Moreover, among all model–RAG combinations, RAG‐Tan + Opus scored the highest (74.9 ± 13.6) (Figure 2B; and Table S1, Supporting Information), suggesting that LLMs with long text–processing capabilities combined with RAG that return more vectors can achieve better overall performance compared to LLMs without these features.

The above analysis revealed clear patterns in how different models respond to varying RAG return volumes. The claude‐3‐opus‐20240229 model showed a consistent positive association between performance and RAG input volume, achieving its highest score with RAG‐Tan. This combination (Claude + RAG‐Tan) led to significant improvements compared to RAG5 in terms of scientific accuracy (16.8 ± 2.1 vs 13.2 ± 2.4 out of a possible maximum score of 20; *p* < 0.01) and completeness (7.2 ± 1.1 vs 5.4 ± 1.3 out of a possible maximum score of 10; *p* < 0.001). By contrast, the GPT‐3.5‐turbo‐0125 and GPT‐3.5‐turbo‐16k models reached their highest performance at moderate RAG return volumes, with their scientific accuracy scores peaking with RAG10 (14.5 ± 1.8) or RAG15 (14.8 ± 1.9), respectively, before declining with larger retrieval volumes (Figure S4 and Table S6, Supporting Information).

Like claude‐3‐opus, the GPT‐4o model demonstrated continuous improvement with increasing RAG input. Particularly noteworthy was its performance in complex reasoning tasks, where RAG‐Tan + 4o achieved significantly higher depth scores (7.8 ± 1.2 out of a possible maximum of 10) than did lower RAG volumes. Certain evaluation dimensions, such as references (4.1 ± 0.6 out of a possible 5) and language quality (4.2 ± 0.5 out of a possible 5) remained relatively stable across different RAG volumes, suggesting that these dimensions depend more on the capability of the base model than on retrieval volume (Figure S4 and Table S6, Supporting Information).

### 2.3 Fine‐Tuning PlantGPT for Enhanced Performance

**Figure 3:**
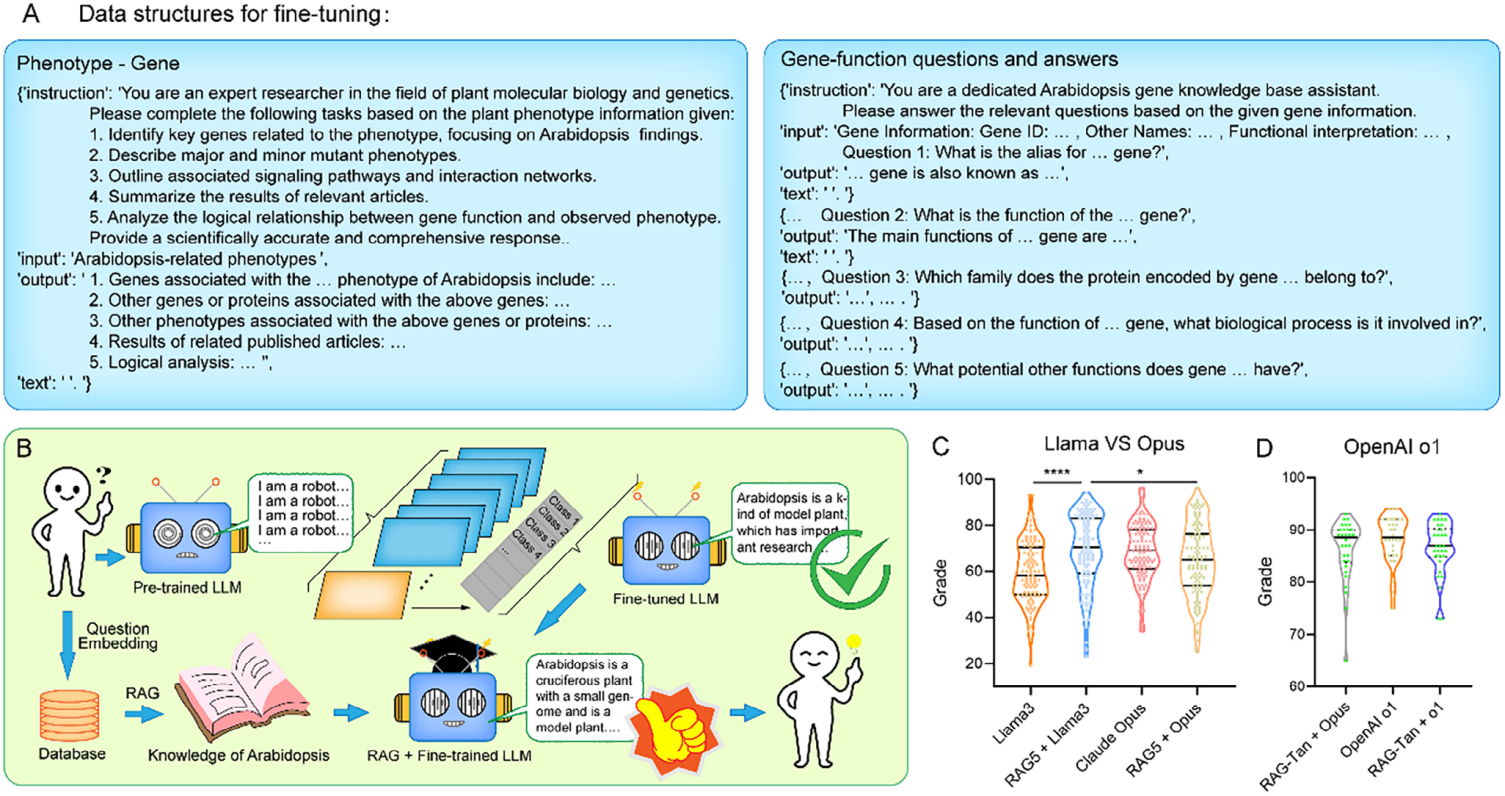
Structure and training effects of the PlantGPT pretraining corpus. A) Structure of the Arabidopsis pre‐training knowledge database used to train Llama3, with phenotype–gene‐related knowledge structure (left) and gene function question–answer pairs (right). B) Enhancing answer quality about specialized knowledge by fine‐tuning with a corpus of Arabidopsis question–answer pairs. C) Scoring results of the ability to answer specialized knowledge questions using both fine‐tuned Llama3 and (the base) Claude Opus. Data are presented as mean ± SD, *n* = 110. D) Scoring results for 10 inferential questions. 30 scores (*n* = 30) per model. *p*‐values were calculated using unpaired two‐tailed *t*‐test, *p < 0.05, **p < 0.01, ***p < 0.001, ****p < 0.0001. “ns” indicates no significant difference.

Building upon the insights from the RAG evaluations described above, we wished to enhance model performance through fine‐tuning. To create a basic model of question–answer pairs about plant functional genomics information, we used gene data gathered from research in Arabidopsis (https://www.arabidopsis.org and http://rarge.gsc.riken.jp/trait) to generate 101 000 phenotype–gene and gene–function relationship question–answer pairs using general‐purpose LLM. We used these pairs to fine‐tune the Meta Llama3 8B open‐source LLM, enabling the resulting Arabidopsis‐Llama3 to answer questions requiring expertise in Arabidopsis‐related molecular biology (**Figure** **3A,B**; and Table S1, Supporting Information). However, due to the length limitations of Llama3 8B in terms of stability and text processing (https://ai.meta.com/blog/meta‐llama‐3/), we were only able to stream the RAG5 results before utilizing Arabidopsis‐Llama3 to enhance its question–answering capabilities.

We tested the above fine‐tuned model with our set of 110 questions. We established that RAG5+Llama3‐fine‐tuned (69.3 ± 15.7 out of a possible maximum score of 100) could answer Arabidopsis‐specific questions more accurately and in more detail than Llama3‐Arab (59.4 ± 13.35) (Figure 3C; and Tables S1 and S5, Supporting Information). RAG5+Llama3‐fine‐tuned also performed slightly better than Claude Opus (68.45 ± 12.3) and RAG5 + Opus (64.7 ± 15.5) (Figure 3C). These results demonstrate that the integrated use of complementary Arabidopsis‐related linguistic data from different sources (literature abstracts and database annotations) for enhanced retrieval and fine‐tuning can achieve a cumulative effect, enhancing the ability of a model to answer specialized questions and increasing corpus utilization.

To gain deeper insights into model performance, we conducted a detailed analysis of response patterns across different question types. We divided all scored questions exceeding mean ± SD (64.8 ± 15.8) into a set of 31 high‐scoring questions and a set of 23 low‐scoring questions for comparison (Figure S1 and Table S2, Supporting Information). In terms of content, the high‐scoring questions asked fundamental questions with high‐confidence answers deeply supported by empirical data, often asking “What is it?” or “What is its function?”. The low‐scoring questions included more complex questions about cutting‐edge research, possibly involving controversial and not fully supported or accepted conclusions, often asking “How?,” “Why?,” or “What happens under specific conditions?”. Regarding the type of question in the two sets, questions in the high‐scoring set were more direct and precisely worded, used simple standard scientific terms, and focused more on Arabidopsis. Low‐scoring questions often included multiple subquestions requiring complex reasoning, possibly because a broader range of answers and complex terminology may limit the effectiveness of RAG retrieval or even lead to errors^[^ ^33^ ^]^ (Table S2, Supporting Information). In summary, the use of concise prompts to obtain a direct and simple answer can yield better results, while complex inferential questions may be more challenging to answer satisfactorily.

For complex reasoning tasks, we developed a separate evaluation framework and employed multiple advanced LLMs (claude‐3‐5‐sonnet‐20241022, GPT‐4o, and GPT‐4 o1‐preview) as evaluators to ensure a comprehensive and reliable assessment. This modified framework emphasizes logical reasoning while maintaining high standards for knowledge accuracy, allocating 30% to knowledge accuracy, 25% to logical reasoning, 20% to completeness, 15% to application ability, and 10% to clarity of expression. To systematically evaluate responses, we developed a 100‐point assessment framework comprising five key dimensions: knowledge accuracy (30 points), logical reasoning (25 points), completeness (20 points), application ability (15 points), and clarity of expression (10 points). Knowledge accuracy assesses domain expertise and scientific precision, ranging from comprehensive understanding (27–30 points) to poor comprehension with major errors (0–14 points). Logical reasoning evaluates the clarity of scientific arguments and logical flow, ranging from exceptional reasoning (22–25 points) to invalid connections (0–9 points). Completeness examines the coverage of relevant aspects, ranging from comprehensive (18–20 points) to highly incomplete (0–8 points). Application ability measures the practical implementation of theoretical knowledge, ranging from excellent (13–15 points) to insufficient (0–3 points). Clarity of expression evaluates communication effectiveness, from crystal clear scientific language (9–10 points) to unclear expression (0–2 points). This comprehensive scoring system ensures thorough evaluation of both content quality and presentation effectiveness (Table S7, Supporting Information). Each criterion was carefully weighted based on its importance for scientific reasoning and aligned with recent advances in LLM evaluation methodologies.^[^ ^29^ ^]^

Using this framework, we evaluated model performance on 10 Arabidopsis‐related inferential questions using claude‐3‐opus‐20240229 and OpenAI's o1‐preview (Figure 3D; and Table S1, Supporting Information). The o1‐preview model demonstrated superior reasoning ability and stability, performing slightly better than RAG‐Tan + Opus, while RAG‐Tan + o1 fared slightly worse than o1‐preview (Table S1, Supporting Information). However, the answers given by o1‐preview contained many hallucinations, which were corrected after integrating RAG into the model (Tables S1 and S8, Supporting Information). A focused analysis of the logical reasoning metric showed no difference in reasoning ability between o1‐preview and RAG‐Tan + o1 (Figure S2, Supporting Information), although incorporating RAG‐Tan into the o1‐preview model resulted in an overall lower score than that for answers given by o1‐preview alone (Table S7, Supporting Information). This outcome may be due to the use of chain‐of‐thought prompting by o1‐preview, making it difficult to integrate prompt engineering and RAG (https://openai.com/index/learning‐to‐reason‐with‐llms/).

### 2.4 Cross‐Species Generalization Capability of PlantGPT

Having established the strong performance of PlantGPT for Arabidopsis‐specific tasks, we assessed its ability to generalize Arabidopsis‐centric knowledge to major crop species, which is crucial for broader agricultural applications. We designed a test set of 30 standardized questions (10 each for rice [*Oryza sativa*], wheat [*Triticum aestivum*], and maize [*Zea mays*]) covering key aspects of plant functional genomics (Table S9, Supporting Information). To systematically assess the cross‐species applicability of PlantGPT, we established a comprehensive 100‐point scoring system encompassing four key dimensions: response accuracy (35 points), information completeness (25 points), reasoning quality (20 points), and transfer and generalization (20 points). Response accuracy (35 points) evaluates descriptions of gene function (10 points), phenotypic effects (10 points), molecular mechanisms (10 points), and literature citations (5 points). Information completeness (25 points) assesses research background (5 points), regulatory networks (10 points), and application value (10 points). Reasoning quality (20 points) examines logical reasoning (10 points) and information integration (10 points). Transfer and generalization (20 points) focuses on knowledge transfer from Arabidopsis to crops (10 points), evolutionary conservation analysis (5 points), and hypothesis generation (5 points). Each subcategory is scored based on the quality and depth of information provided, from basic descriptions (2–4 points) to comprehensive analyses (8–10 points). This scoring system ensures the capability of PlantGPT to effectively translate Arabidopsis knowledge to crop research applications (Table S10, Supporting Information).

**Figure 4:**
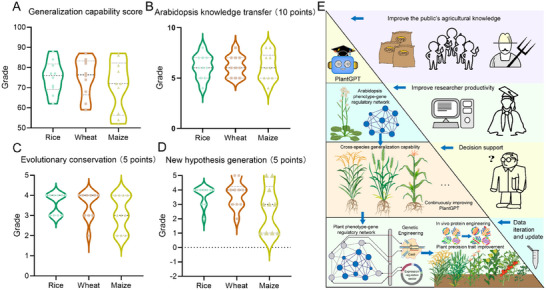
Cross‐plant species evaluation of PlantGPT performance. A) Overall generalization‐capability scores, on a 100‐point scale. B) Score of knowledge transfer from Arabidopsis to rice, wheat, or maize, on a 10‐point scale. C) Evolutionary‐conservation scores in rice, wheat, and maize, on a 5‐point scale. D) New hypothesis‐generation scores in rice, wheat, and maize, on a 5‐point scale. Data are presented as mean ± SD, *n* = 10. The values in the violin plots are shown in Table S10 (Supporting Information). E) Framework diagram of the knowledge transfer system and applications of PlantGPT.

Overall, we observed strong generalization capabilities across all three crops (**Figure** **4**A), with mean scores of 75.1 ± 8.0 for rice, 75.2 ± 9.5 for wheat, and 71.4 ± 12.9 for maize. Rice showed particularly robust performance in terms of response accuracy (30.2 out of a possible 35 points), while wheat demonstrated strong information completeness (21.3 out of a possible 25 points). The performance differences among crops highlight the model's ability to effectively process diverse plant genomic data, although the specific factors contributing to these variations merit further investigation.

Following our evaluation approach for inferential questions (Table S10, Supporting Information), we employed GPT‐4 (o1‐preview) to evaluate responses to standardized questions about rice, wheat, and maize using this detailed rubric. The knowledge transfer capability of PlantGPT showed remarkable consistency across species (Figure 4B), with scores of 6.1 ± 1.2 (rice), 6.1 ± 1.0 (wheat), and 6.1 ± 1.4 (maize) out of a possible 10 points, particularly for understanding pathway conservation and for transferring functional annotations. This uniform performance suggests robust cross‐species applicability of Arabidopsis‐derived knowledge. An analysis of evolutionary conservation (Figure 4C) revealed a gradient consistent with known phylogenetic relationships (3.6 ± 0.5 [rice], 3.5 ± 0.7 [wheat], 3.1 ± 0.9 [maize] out of a possible 5 points), indicating that PlantGPT's predictions mirror both the depth of existing research and the degree of data correlation between different plant species.

Notably, PlantGPT exhibited strong hypothesis‐generation capabilities (Figure 4D) across all species (3.6 ± 0.7 [rice], 3.7 ± 0.9 [wheat], 2.7 ± 1.6 [maize] out of a possible 5 points), suggesting effective translation of Arabidopsis research paradigms to crop species. The model consistently provided testable predictions and experimental approaches, with particularly strong performance in regulatory network inference and functional conservation analysis (Table S10, Supporting Information).

The scoring system showed appropriate discriminative ability, with scores ranging from 52 to 88 (mean = 73.7; SD = 10.2), effectively differentiating between varying levels of response quality. These results demonstrate that despite its Arabidopsis‐centric training set, PlantGPT successfully generalizes functional genomics knowledge across diverse crop species, with variation in performance primarily reflecting known evolutionary relationships.

## 3 Discussion

In this study, we determined that our newly developed RAG+LLM‐based tool PlantGPT can achieve better responses than Claude Opus with a relatively low RAG input (Figure 3C). Our comprehensive evaluation framework, encompassing both specialized knowledge and inferential reasoning capabilities, provides robust evidence for the effectiveness of our approach. Combining RAG and fine‐tuning enhanced the focus on the Arabidopsis research field, enabling the model to utilize information more accurately and efficiently while diminishing interference from irrelevant information.^[^ ^34^ ^]^ Whereas Gao et al.^[^ ^33^ ^]^ provided a broad survey of RAG for LLMs, our study focused on the practical applications of LLMs for Arabidopsis research. For Arabidopsis drought response queries, Arabidopsis‐Llama3 provided professional, reliable knowledge with hallucination‐reduction references, outperforming Claude Opus in providing timely data and aiding researchers in finding research entry points. The performance of RAG10 + GPT3.5‐turbo‐0125 was comparable to that of Claude Opus (Table S3, Supporting Information). We also achieved satisfactory results for specialized questions about gene function and interactions, including molecular mechanisms and functional associations in biological systems (Table S4, Supporting Information).

The observed variation in terms of which RAG configuration offers the best outcome across different model scales provides important insights for practical implementations. While larger LLMs such as Claude Opus and GPT‐4 benefit from larger retrieval volumes, the performance peak of smaller models at moderate RAG input levels (RAG10 or RAG15) suggests a crucial balance between information richness and processing capacity. This finding extends beyond previous work by Zhao et al.,^[^ ^35^ ^]^ who surveyed RAG for artificial intelligence (AI)‐generated content, by providing empirical evidence for its effectiveness specifically in plant genomics. Our approach of combining a specialized vector database with RAG notably enhances answer accuracy and quality for domain‐specific questions when using models like GPT‐3.5‐turbo‐0125, offering cost‐effective problem‐solving options and facilitating deeper reasoning. This implementation goes beyond the general synergy discussed by Feng et al.,^[^ ^36^ ^]^ showing specific benefits for plant research.

Particularly noteworthy is the strong performance of PlantGPT in cross‐species knowledge transfer. The graded performance across rice, wheat, and maize, which reflects their evolutionary distances from Arabidopsis, demonstrates the ability of this model to leverage fundamental biological principles while accounting for species‐specific variations. This capability is especially valuable for translating research insights from model organisms to crop species. Furthermore, the consistent performance in hypothesis generation and understanding pathway conservation across different crops suggests that the PlantGPT model boasts a robust framework for biological knowledge generalization. The standardized and modular nature of our vector database implementation supports this cross‐species applicability, making it easy to update and ensure the accurate representation of research progress for various plant species in real time. The broad applicability of PlantGPT is manifest in its multitiered impacts: facilitating public access to agricultural knowledge, accelerating knowledge acquisition by early‐career researchers, and supporting strategic decision‐making by experienced investigators. This versatility, coupled with its continuous update mechanism, enhances both research efficiency and knowledge dissemination in the plant sciences (Figure 4E).

While our current RAG technology has limitations in accurately retrieving key information, in the future, we plan to adopt advanced technologies such as HippoRAG,^[^ ^37^ ^]^ GraphRAG for improved relationship‐based retrieval,^[^ ^38^ ^]^ and modern vector databases including Milvus (https://milvus.io, https://github.com/milvus‐io/milvus). Inspired by the innovative small‐parameter but high‐performance reasoning paradigm of DeepSeek, we envision developing efficient and cost‐effective approaches for inferring complex gene–phenotype knowledge (https://api‐docs.deepseek.com/). We aim to expand our focus to multiomics research, enabling multimodal tasks and thought chains to assist in predicting gene function and experimental decision‐making, combined with cutting‐edge bioinformatics analysis technologies.^[^ ^39^, ^40^ ^]^

In conclusion, we developed PlantGPT to answer plant domain–specific knowledge questions by combining vector databases and RAG with general LLMs, with a focus on Arabidopsis. This approach provides answers with minimal hallucinations and effective information for functional genomics reasoning at a low cost. PlantGPT will help scientists quickly enter new fields of research in plant genomics and improve the impact of this research and offer references for the mapping of regulatory networks and for functional genomics research in crops. Building on our successful results for cross‐species generalization, we plan to extend this high‐quality answering system to a broader range of crop species, particularly focusing on yield‐related traits and stress responses, which are crucial for agricultural advancements.

## 4 Experimental Section

### Preprocessing of Arabidopsis Phenotype–Gene Data

The Arabidopsis‐related gene and phenotype data primarily originated from structured data files available in TAIR (https://www.arabidopsis.org). Phenotypes mentioned in the Locus_Published_20 230 407.txt (structured data file) were preprocessed using Anthropic's general‐purpose LLM claude‐3‐sonnet‐20240229, obtaining 14 000 Arabidopsis‐related phenotypes. Subsequently, claude‐3‐opus‐20240229 was used to process these 14000+ phenotypes, extracting information along six points of focus: functional genes, related genes, associated phenotypes, article sources, logical reasoning, and important homologous genes in crops. This information was structured into logical question–answer pairs. Fine‐tuning of the database was designed based on these question–answer pairs to enhance the systematic understanding of Arabidopsis gene research by Llama3 8b. Over 23 000 reported Arabidopsis gene IDs were downloaded from TAIR with their corresponding former names when applicable and their functional annotations. Using claude‐3‐5‐sonnet‐20240620, question–answer pairs were generated for these gene IDs covering five aspects: gene aliases, gene functions, gene family information, known biological processes in which they are involved, and potential biological processes in which they are involved (inferential questions). Question–answer pairs from these two sources were used for fine‐tuning. The Arabidopsis data corpus used for fine‐tuning can be found at https://drive.google.com/file/d/1cam5m0Ty0lCrbnvhirn_IMArcu4DLReB/view?usp=haring.

### Fine‐Tuning and Downstream Tasks

Our fine‐tuning tasks primarily focused on purely downstream text tasks. Specifically, a question–answer pair structure was used for fine‐tuning (Figure 2A). For a description of the fine‐tuning steps, please refer to Unsloth (https://github.com/unslothai/unsloth.git). For downstream tasks, claude‐3‐5‐sonnet‐20240620 was used to refine the language of the responses by the Llama3 model to ensure language stability.

### Vector Database Establishment and RAG

A PubMed search for articles containing the keyword “Arabidopsis” published between 1992 and 2024 yielded over 70 000 results. To ensure the quality and breadth of the corresponding knowledge base, a multilayered journal selection procedure was implemented. Specifically, JCR quartile rankings and Impact Factors in Natural Sciences (June 2024, Clarivate Analytics) were accessed, with a specific focus on plant sciences (Table S5, Supporting Information). Journals ranked within the Q1–Q3 quartiles and the top 60% in the field were chosen as the primary target. For multidisciplinary journals (*Nature*, *Science*, *Cell*, and similar journals), separate evaluation criteria were applied, considering their unique contributions to Arabidopsis research. Additionally, the historical impact and long‐term performance of individual journals to plant sciences were considered, particularly those focused on plant molecular biology and functional genomics. Using these rigorous criteria, a set of 60 429 high‐quality Arabidopsis research articles was obtained.

To establish a reliable vector database, data deduplication was performed using a two‐step approach to ensure data quality and temporal relevance. First, duplicate entries were identified through exact matching of Digital Object Identifiers and PubMed IDs. Second, when similar abstracts were identified, preference was given to the more recent publication to maintain the temporal currency of the research findings. This systematic deduplication process helped establish a reliable, up‐to‐date vector database while preserving the chronological relevance of the included studies. To address the text‐processing capacity limitations of AI language models and their associated computational costs, an optimal approach for abstract corpus segmentation was explored. Language models have a maximum limit on the amount of text they can process at once (similar to an upper bound on how much text they can read in a single instance). Segmenting by entire abstracts led to redundancy, decreasing the precision of retrieval‐augmented generation systems and unnecessarily burdening the model with excessive text to process.

To determine the optimal granularity for abstract corpus segmentation, different granularity levels were systematically evaluated through comparative analysis using 2000 randomly selected abstracts. Multiple segmentation approaches were evaluated, including complete abstract segmentation, sentence‐based segmentation, fixed token lengths (64 or 128 tokens), and overlapping strategies with varying word counts and overlap lengths (30–10, 50–20, 50–30, and 60–40). The evaluation employed a comprehensive 100‐point scoring system assessing five dimensions: scientific accuracy (20 points), information coverage (25 points), logical coherence (20 points), context preservation (20 points), and response efficiency (15 points). Detailed scoring criteria and results can be found in Table S6 and Figure S3 (Supporting Information).

Following the optimization experiments, a 50–30 strategy (50 words with 30‐word overlap) was implemented for abstract segmentation. The divided sentences formed basic abstract fragments, which were embedded using OpenAI's text‐embedding‐3‐small with 1536‐dimensional embedding (https://help.openai.com/en/articles/8868588‐retrieval‐augmented‐generation‐rag‐and‐semantic‐search‐for‐gpts). The deduplicated embedded vectors were stored in a Chroma database for efficient retrieval. Additionally, all reported Arabidopsis gene IDs and their previously held names were vectorized, storing them alongside the abstract embeddings. To ensure that the database is up‐to‐date and comprehensive, a 3‐month (quarterly) update mechanism was established.

To evaluate RAG retrieval, different retrieval volumes were tested using a gradient approach: retrieving the top five (RAG5), top 10 (RAG10), top 15 (RAG15), top 20 (RAG20), and all vectors before the inflection point of the similarity vector return value (RAG‐Tan). These different retrieval strategies were evaluated for their answers to 110 Arabidopsis‐specific questions. The responses were systematically assessed using 12 criteria, including accuracy, completeness, and context preservation, with weighted scores assigned according to their importance (Table S1, Supporting Information). The vector database and all evaluation datasets are available at https://drive.google.com/file/d/1–55JEkF9u3NGCoVZyyx2SOti7TGPL0S/view?usp=sharing.

### Test Evaluation Metrics

Primarily testing questions related to Arabidopsis research were focused on, while also conducting extensibility tests on questions about other crops to validate the broader applicability of the research. The metrics used for testing can be found in Tables S3, S4, S6, S8, and S10 (Supporting Information). Evaluation criteria for inferential questions are listed in Table S4 (Supporting Information). The mean ± SD for the score obtained from 2640 questions was calculated, reaching 64.8 ± 15.8, with scores falling outside this range (≤49.0 or ≥80.6) defined as extreme scores. Among the 24 models, each model was tested at least five times. Since each model was tested at least five times on every question, extreme questions were defined as those receiving five or more extreme scores. This threshold ensured that flagged questions consistently triggered model instability across repeated evaluations, rather than reflecting random anomalies in single test runs (Table S2, Supporting Information).

### Computational Resources and Implementation Details

The study used various computational resources across different stages of implementation. For the initial embedding stage and vector database construction, OpenAI's text‐embedding‐3‐small model was used to embed the abstracts of 60 000 research articles, which were segmented into ≈600 000 text segments. This embedding step was performed on a computer with 64 GB RAM (although testing confirmed its functionality to be an effective 32 GB RAM) running Python 3.13.0 and ChromaDB (version 0.4.22). The embedding step cost ≈US$0.78 in computational needs, and the resulting vectors were stored in a ChromaDB database for efficient retrieval.

The model fine‐tuning phase was conducted in the Google Colab environment, using an NVIDIA Tesla T4 GPU with 14.748 GB VRAM. The software environment comprised PyTorch 2.4.0+cu121, Transformers 4.44.2, CUDA 7.5, and CUDA Toolkit 12.1, with Xformers 0.0.27.post2 used for optimization. Under standard Colab configurations, users can utilize the NVIDIA Tesla T4 GPU for ≈3 h and 45 min without additional purchases, which proved sufficient to complete the fine‐tuning process. For extended computational needs, an optional 200 Compute Unit purchase (costing US$20.98) is available through the Colab platform to ensure uninterrupted training sessions. The fine‐tuning step was completed in ≈1 h, following the implementation guidelines from the Unsloth (https://github.com/unslothai/unsloth.git) repository.

For model inference and deployment, a high‐performance computing system was used that runs Windows 10 (version 17 763), equipped with an AMD EPYC 7542 32‐Core Processor (2895 MHz), 64 GB RAM, and an NVIDIA GeForce RTX 2080 Ti GPU. The inference system was implemented using LM Studio (version 0.3.5) within a VMware 20.1 virtualization environment. This configuration processed the test set of 110 questions in ≈55 min, demonstrating efficient real‐time performance for practical applications.

Throughout the development and testing phases, extensive use was made of Application Programming Interface (API) services from OpenAI and Anthropic, totaling 268 064 API calls for a combined cost of ≈US$1290. This comprehensive setup enabled to develop and validate Plant‐GPT effectively, ensuring both performance and reliability for plant science research applications. All code and implementation details are available on the GitHub repository to facilitate reproducibility and further development by the research community.

The computational resources and implementation details described above represent the minimum requirements for reproducing the results. Researchers using similar systems might need to adjust their hardware and software configurations based on their specific requirements and scale of implementation.

### Statistical Analysis

Continuous variables are expressed as mean ± standard error of the mean. For scoring experiments involving 110 Arabidopsis‐related knowledge questions (*n* = 110), 10 inferential questions with 30 total scores (*n* = 30), and 10 questions evaluating Arabidopsis knowledge transfer capability (*n* = 10), normality tests were conducted to ensure the data met the assumptions for parametric testing. Group comparisons were performed using unpaired two‐tailed *t*‐tests. In all analyses, *p* < 0.05 was considered statistically significant (*p < 0.05, **p < 0.01, ***p < 0.001, ****p < 0.0001). Statistical analysis was conducted using GraphPad Prism 10.1.2(324) software (GraphPad Software Inc., San Diego, CA).

## Conflict of Interest

The authors declare no conflict of interest.

## Supporting information



Supporting Information

Supplemental Table 1

## Data Availability

The data that support the findings of this study are available in the supplementary material of this article.
