# Supplementary material for: PlantGPT: An Arabidopsis‐Based Intelligent Agent that Answers Questions about Plant Functional Genomics
Source: Adv Sci (Weinh). 2025 May 21;12(30):e03926. doi: 10.1002/advs.202503926 (PMC12376578; doi:10.1002/advs.202503926)
Supplement: Supplementary file 1 — Supporting Information [file ADVS-12-e03926-s002.docx]

Supplementary data

**1. Prompt for Llama3 language polish**

Please polish and optimize the text of your answers to the following Arabidopsis-related questions:

1. Remove repetitive or redundant content and keep the core information.

2. Optimize the language expression and use accurate and professional terms.

3. Enhance the logic and ensure the coherence between the various points.

4. Make sure each paragraph has a clear central idea.

5. Make sure the answer is directly addressed to the question and does not deviate from the topic.

6. Do not answer any unnecessary content!

Supplementary Table 1: Whitelist of Selected Articles

Supplementary Table 2: Questions and Answers After Sentence-level Granularity Segmentation

Supplementary Table 3: Granularity Scoring Criteria

Supplementary Table 4: Scoring Criteria for 110 Questions

Supplementary Table 5: 110 Questions and Answers

Supplementary Table 6: Mean ± SD of RAG Performance on Different Questions

Supplementary Table 7: Inferential Questions Scoring

Supplementary Table 8: Inferential Questions and Answers

Supplementary Table 9: Extension Questions and Answers

Supplementary Table 10: Extension Questions and Scoring

Extended Figure 1. Extreme questions rating heat map.


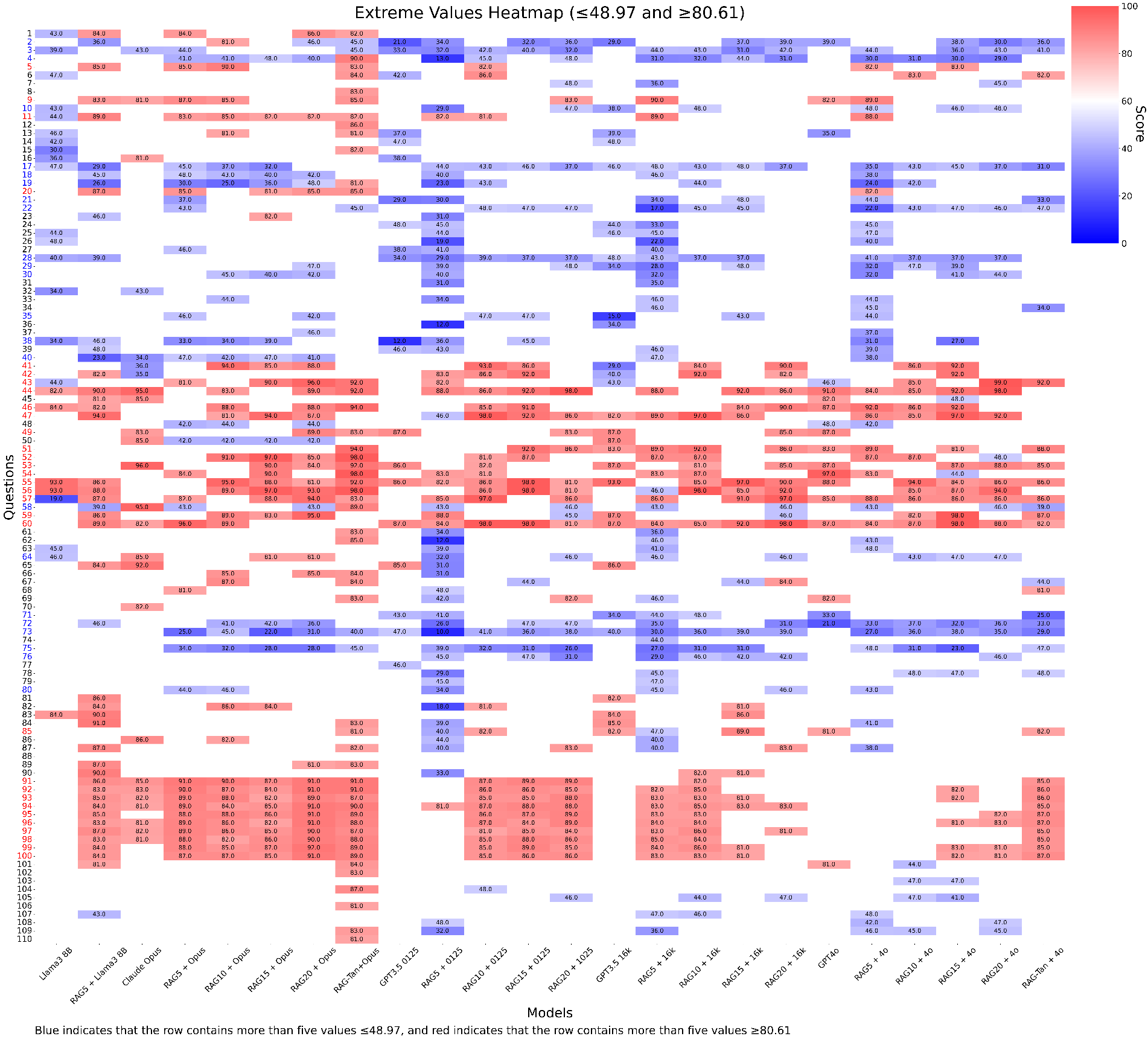


Extended Figure 2. Score of logical reasoning ability for inference problems (out of 25)


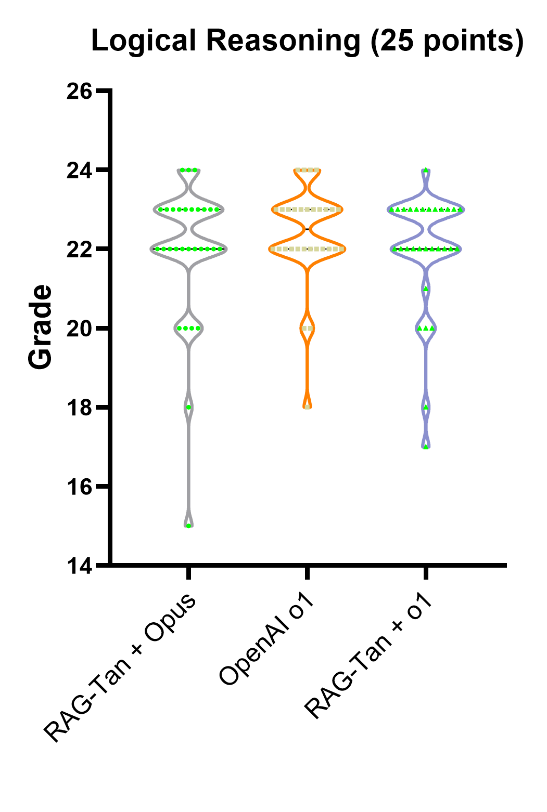


Data are presented as mean ± SD, n=30.

Extended Figure 3. The impact of sentence granularity segmentation on answer quality


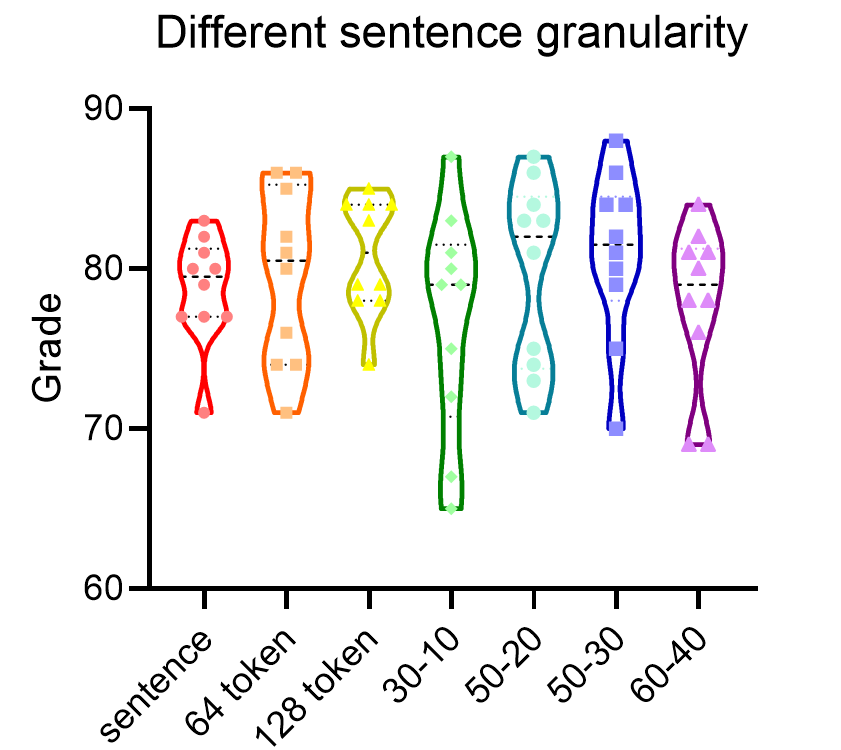


Data are presented as mean ± SD, n=10.

Extended Figure 4. Specific impact of RAG return volume


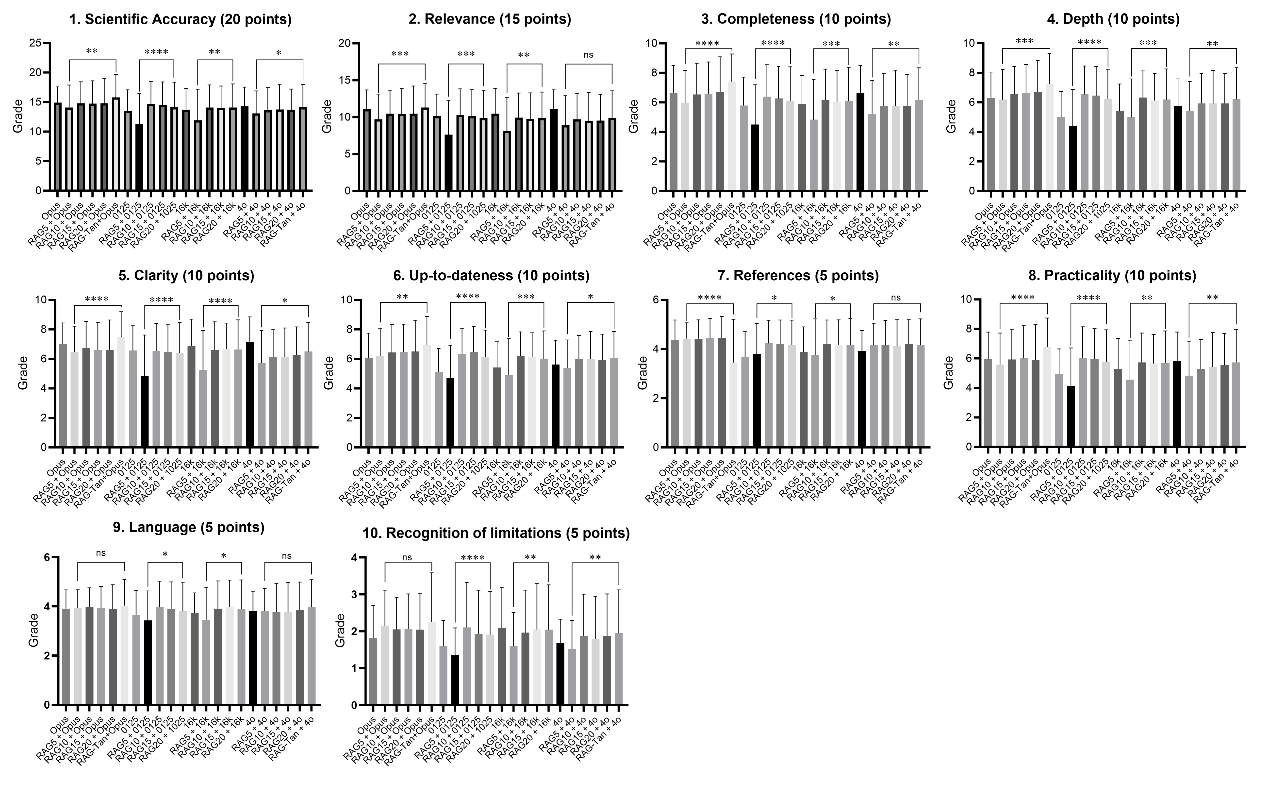


Data are presented as mean ± SD, n=110. P-values were calculated using unpaired two-tailed t-test, **P*<0.05, ***P*<0.01, ****P*<0.001, *****P*<0.0001. "ns" indicates no significant difference.

Extended Table 1. Sample questions for all model tests with mean scores and standard deviations (SD)

| Model | Answer sample | Mean ± SD |
| --- | --- | --- |
| Llama3 8B | Query: 1. Does *Arabidopsis* reproduce by self-pollination or hybridization? *Arabidopsis* *thaliana* is predominantly self-pollinating. This reproductive strategy offers several advantages:  1. Genetic stability: Self-pollination maintains homozygosity, preserving beneficial gene combinations.  2. Rapid life cycle: Self-compatibility enables quick seed production, facilitating multiple generations per year.  3. Research efficiency: Self-pollination simplifies genetic studies by producing genetically uniform offspring.  4. Resource conservation: The plant doesn't require pollinators, conserving energy for seed production.  5. Colonization ability: Self-pollination allows a single plant to establish new populations in isolated areas.  However, this strategy also limits genetic diversity, potentially reducing adaptability to environmental changes. Despite this, *Arabidopsis*' self-pollinating nature has made it an invaluable model organism in plant genetics and molecular biology research. | 59.39 ± 13.35 |
| RAG5 + Llama3 | Query: 1. Does *Arabidopsis* reproduce by self-pollination or hybridization?  ### Response:  *Arabidopsis* primarily reproduces by self-pollination [1][3][5]. However, it also exhibits a low frequency of insect-mediated outcrossing in wild populations [3][4]. While *Arabidopsis* is capable of self-pollination, there are some mechanisms that can promote outcrossing:  1. The flowers emit monoterpenes and sesquiterpenes, which may serve as olfactory cues for pollinating insects [3].  2. In the early phases of flowering, the stigma cannot be pollinated by its own pollen due to short filaments [4].  These features suggest that while self-pollination is the primary mode of reproduction, *Arabidopsis* has retained some capacity for hybridization through outcrossing.  Reference:  [1] Setting up *Arabidopsis* crosses, CSH Protoc, pmcid: 10.1101/pdb.prot4623  [3] Biosynthesis and emission of terpenoid volatiles from *Arabidopsis* flowers, Plant Cell, pmcid: PMC141215  [4] Transgenic GFP as a molecular marker for approaches to quantify pollination mechanism and gene flow in *Arabidopsis* *thaliana*, Plant Biol (Stuttg), pmcid: 10.1055/s-2005-837734  [5] The evolution of selfing in *Arabidopsis* *thaliana*, Science, pmcid: 10.1126/science.1143153 | 69.31 ± 15.66 |
| Claude Opus | Query: 1. Does *Arabidopsis* reproduce by self-pollination or hybridization?  ### Response:  *Arabidopsis* *thaliana*, the model plant commonly used in research, primarily reproduces by self-pollination (selfing) [1]. Self-pollination occurs when pollen from the anthers of a flower fertilizes the ovules of the same flower or another flower on the same plant. This results in offspring that are genetically identical to the parent plant.  However, *Arabidopsis* is also capable of outcrossing (hybridization) at low frequencies, typically less than 0.3% in the wild [2]. Outcrossing occurs when pollen from one plant fertilizes the ovules of a different plant, resulting in genetically diverse offspring.  References:  [1] Meinke, D. W., Cherry, J. M., Dean, C., Rounsley, S. D., & Koornneef, M. (1998). *Arabidopsis* *thaliana*: a model plant for genome analysis. Science, 282(5389), 662-682. DOI: 10.1126/science.282.5389.662  [2] Abbott, R. J., & Gomes, M. F. (1989). Population genetic structure and outcrossing rate of *Arabidopsis* *thaliana* (L.) Heynh. Heredity, 62(3), 411-418. DOI: 10.1038/hdy.1989.56 | 68.45 ± 12.34 |
| RAG5 + Opus | Query: 1. Does *Arabidopsis* reproduce by self-pollination or hybridization?  ### Response:  *Arabidopsis* *thaliana* is capable of self-pollination, but outcrossing can also occur at low frequencies in wild populations [3,5]. *Arabidopsis* flowers emit monoterpenes and sesquiterpenes, which may act as olfactory cues for pollinating insects [3]. However, *Arabidopsis* is believed to be mostly self-pollinated [3].  Interestingly, the stigma in each *Arabidopsis* flower cannot be pollinated by its own pollen in the early phases of the flowering process, when the anthers have dehisced but the filaments are still too short [4]. This suggests a mechanism to promote outcrossing.  Unlike most of its close relatives in the mustard family, *Arabidopsis* lacks the complex self-incompatibility (S) locus, which harbors multiple diverged specificity haplotypes that effectively prevent selfing [5]. The absence of this locus likely played a role in the evolution of selfing in *Arabidopsis* [5].  To generate cross-progeny in *Arabidopsis*, which naturally self-pollinates, intervention by the investigator is required [1]. This involves setting up crosses between suitable *Arabidopsis* parent plants and collecting the resulting seeds [1].  References:  [1] Setting up *Arabidopsis* crosses, CSH Protoc, PMID: 22485970, DOI: 10.1101/pdb.prot4623  [3] Biosynthesis and emission of terpenoid volatiles from *Arabidopsis* flowers, Plant Cell, PMCID: PMC141215, PMID: 12566586  [4] Transgenic GFP as a molecular marker for approaches to quantify pollination mechanism and gene flow in *Arabidopsis* *thaliana*, Plant Biol (Stuttg), PMID: 16025413, DOI: 10.1055/s-2005-837734  [5] The evolution of selfing in *Arabidopsis* *thaliana*, Science, PMID: 17656687, DOI: 10.1126/science.1143153 | 64.69 ± 15.49 |
| RAG10 + Opus | Query: 1. Does *Arabidopsis* reproduce by self-pollination or hybridization?  ### Response:  *Arabidopsis* *thaliana* is a self-pollinating plant species, meaning that it can produce seeds without the need for cross-pollination between different plants [1, 4, 5, 9]. However, some degree of outcrossing, or cross-pollination, can occur naturally in wild populations, likely mediated by insect pollinators [3, 4].  *Arabidopsis* flowers emit both monoterpenes and sesquiterpenes, which may serve as olfactory cues to attract pollinating insects [3]. Despite this, the stigma of an *Arabidopsis* flower cannot be pollinated by its own pollen in the early stages of flowering, as the filaments are initially too short to allow the anthers to contact the stigma [4]. Successful self-pollination occurs when the stamen and pistil lengths align, allowing pollen from the stamen tip to be delivered to the stigma at the pistil tip [7].  The evolution of self-pollination in *Arabidopsis* *thaliana* is thought to be related to the loss of the complex self-incompatibility (S) locus, which is present in other members of the mustard family and prevents self-pollination [5]. This loss of the S locus likely allowed for the transition from outcrossing to selfing in A. *thaliana* [5, 6].  To generate cross-progeny for genetic studies, researchers must manually cross-pollinate *Arabidopsis* plants, as the natural self-pollination makes it difficult to obtain outcrossed offspring without intervention [1].  References:  [1] Setting up *Arabidopsis* crosses, CSH Protoc, PMID: 22485970, DOI: 10.1101/pdb.prot4623  [3] Biosynthesis and emission of terpenoid volatiles from *Arabidopsis* flowers, Plant Cell, PMCID: PMC141215, PMID: 12566586  [4] Transgenic GFP as a molecular marker for approaches to quantify pollination mechanism and gene flow in *Arabidopsis* *thaliana*, Plant Biol (Stuttg), PMID: 16025413, DOI: 10.1055/s-2005-837734  [5] The evolution of selfing in *Arabidopsis* *thaliana*, Science, PMID: 17656687, DOI: 10.1126/science.1143153  [6] Neutral evolution of synonymous base composition in the Brassicaceae, J Mol Evol, PMID: 17160366, DOI: 10.1007/s00239-005-0274-1  [7] EPFL peptide signalling ensures robust self-pollination success under cool temperature stress by aligning the length of the stamen and pistil, Plant Cell Environ, PMID: 36419209, DOI: 10.1111/pce.14498  [9] A DNA methyltransferase homolog with a chromodomain exists in multiple polymorphic forms in *Arabidopsis*, Genetics, PMCID: PMC1460135, PMID: 9584105 | 67.52 ± 15.60 |
| RAG15 + Opus | Query: 1. Does *Arabidopsis* reproduce by self-pollination or hybridization?  ### Response:  *Arabidopsis* *thaliana* is a self-pollinating plant species, meaning that it can produce seeds through self-fertilization without the need for cross-pollination [1, 4, 5, 9, 11]. However, despite being predominantly self-pollinating, *Arabidopsis* is still capable of outcrossing, which involves the transfer of pollen from one plant to another [3, 4, 8, 12, 13].  The process of self-pollination in *Arabidopsis* requires the alignment of stamen and pistil lengths within a single flower, allowing pollen from the stamen tip to be delivered to the stigma at the pistil tip [7, 11]. This process is regulated by intercellular signaling, including secreted peptides [7]. Successful pollination involves several sequential events, from pollen adhesion onto the stigma surface to pollen tube germination and elongation [2, 14].  *Arabidopsis* flowers emit both monoterpenes and sesquiterpenes, which may serve as potential olfactory cues for pollinating insects, suggesting that insect-mediated outcrossing can occur at a low frequency in wild populations [3]. Outcrossing can introduce genetic diversity and has implications for the evolution of selfing in *Arabidopsis* [5, 8, 12].  Generating cross-progeny in *Arabidopsis* requires intervention by the investigator, as the plant naturally self-pollinates [1, 10]. This can be achieved by fertilizing flowers side-by-side with either pollen from the same accession (self-pollination) or pollen from another accession (outcrossing) [13].  References:  [1] Setting up *Arabidopsis* crosses, CSH Protoc, PMID: 22485970, DOI: 10.1101/pdb.prot4623  [3] Biosynthesis and emission of terpenoid volatiles from *Arabidopsis* flowers, Plant Cell, PMCID: PMC141215, PMID: 12566586  [4] Transgenic GFP as a molecular marker for approaches to quantify pollination mechanism and gene flow in *Arabidopsis* *thaliana*, Plant Biol (Stuttg), PMID: 16025413, DOI: 10.1055/s-2005-837734  [5] The evolution of selfing in *Arabidopsis* *thaliana*, Science, PMID: 17656687, DOI: 10.1126/science.1143153  [7] EPFL peptide signalling ensures robust self-pollination success under cool temperature stress by aligning the length of the stamen and pistil, Plant Cell Environ, PMID: 36419209, DOI: 10.1111/pce.14498  [8] Mutational meltdown in selfing *Arabidopsis* lyrata, Evolution, PMID: 23461329, DOI: 10.1111/j.1558-5646.2012.01818.x  [11] The AtXTH28 gene, a xyloglucan endotransglucosylase/hydrolase, is involved in automatic self-pollination in *Arabidopsis* *thaliana*, Plant Cell Physiol, PMCID: PMC2642609, PMID: 19139039  [12] Recombination and gene conversion in a 170-kb genomic region of *Arabidopsis* *thaliana*, Genetics, PMCID: PMC1462186, PMID: 12136029  [13] Paternal effects on seed mass in *Arabidopsis* *thaliana*, Plant Biol (Stuttg), PMID: 21134089, DOI: 10.1111/j.1438-8677.2009.00287.x  [14] Transcriptional characteristics and differences in *Arabidopsis* stigmatic papilla cells pre- and post-pollination, Plant Cell Physiol, PMID: 25527828, DOI: 10.1093/pcp/pcu209 | 68.05 ± 15.41 |
| RAG20 + Opus | Query: 1. Does *Arabidopsis* reproduce by self-pollination or hybridization?  ### Response:  *Arabidopsis* *thaliana* is a highly self-pollinating plant species [5,18]. Self-pollination occurs when the stamen and pistil lengths are aligned in a single flower, allowing pollen from the anthers to be delivered to the stigma [7]. This process is facilitated by genes such as AtXTH28, a xyloglucan endotransglucosylase/hydrolase involved in automatic self-pollination [17].  Despite its predominantly selfing nature, *Arabidopsis* is capable of outcrossing [3,4,5]. Flowers emit monoterpenes and sesquiterpenes, which may serve as olfactory cues for pollinating insects [3]. However, the stigma cannot be pollinated by its own pollen in the early phases of flowering when the filaments are still too short [4].  Interspecific and intergeneric hybridization is possible in *Arabidopsis* but often encounters pre-zygotic and post-zygotic barriers [10,12,14]. These barriers can be overcome by manipulating receptor-like kinases and their ligands on the stigma surface [10,15]. Hybridization can lead to changes in mutation rates and genome integrity [13].  To study the inheritance of mutant phenotypes or generate genetic diversity, manual cross-pollination is required [1]. This involves intervening in the natural self-pollination process and carefully transferring pollen from one parent to the stigma of another [1,2].  References:  [1] Setting up *Arabidopsis* crosses, CSH Protoc, PMID: 22485970  [3] Biosynthesis and emission of terpenoid volatiles from *Arabidopsis* flowers, Plant Cell, PMCID: PMC141215  [4] Transgenic GFP as a molecular marker for approaches to quantify pollination mechanism and gene flow in *Arabidopsis* *thaliana*, Plant Biol (Stuttg), PMID: 16025413  [5] The evolution of selfing in *Arabidopsis* *thaliana*, Science, PMID: 17656687  [7] EPFL peptide signalling ensures robust self-pollination success under cool temperature stress by aligning the length of the stamen and pistil, Plant Cell Environ, PMID: 36419209  [10] Antagonistic RALF peptides control an intergeneric hybridization barrier on Brassicaceae stigmas, Cell, PMID: 37806310  [12] Heterochromatin, small RNA and post-fertilization dysgenesis in allopolyploid and interploid hybrids of *Arabidopsis*, New Phytol, PMCID: PMC3756494  [13] Hybridization alters spontaneous mutation rates in a parent-of-origin-dependent fashion in *Arabidopsis*, Plant Physiol, PMCID: PMC4012600  [14] Dissection of two major components of the post-zygotic hybridization barrier in rice endosperm, Plant J, PMID: 24286595  [15] Generation of self-incompatible *Arabidopsis* *thaliana* by transfer of two S locus genes from A. lyrata, Science, PMID: 12114625  [17] The AtXTH28 gene, a xyloglucan endotransglucosylase/hydrolase, is involved in automatic self-pollination in *Arabidopsis* *thaliana*, Plant Cell Physiol, PMCID: PMC2642609  [18] Recombination and gene conversion in a 170-kb genomic region of *Arabidopsis* *thaliana*, Genetics, PMCID: PMC1462186 | 69.00 ± 16.38 |
| RAG – Tan + Opus | Query: 1. Does *Arabidopsis* reproduce by self-pollination or hybridization?  ### Response:  Based on the provided knowledge, here is a summary of key points about pollination and hybridization in *Arabidopsis*:  *Arabidopsis* *thaliana* is a highly selfing species that evolved from outcrossing ancestors through the loss of self-incompatibility [5,26,30]. Despite being predominantly self-pollinating, some natural populations exhibit variation in outcrossing rates [23,28]. Flowers of A. *thaliana* emit volatile compounds that may attract insect pollinators in the wild [3].  The transition from outcrossing to selfing likely involved pseudogenization of the S-locus receptor kinase (SRK) and S-locus cysteine-rich (SCR) genes that control self-incompatibility [15,42]. Related outcrossing species like A. lyrata and A. halleri have functional SI systems [25,33,46].  Interspecific crosses between A. *thaliana* and relatives often result in hybrid incompatibility. Endosperm over-proliferation and seed abortion are common outcomes, similar to interploidy crosses [14,34,44]. Defects may arise from epigenetic deregulation and activation of transposable elements in the hybrid endosperm [12,47].  Nonetheless, viable hybrids can be obtained between A. *thaliana* and some relatives [13,35,36,38]. Hybrid vigor for traits like photosynthesis and growth has been observed in certain crosses [35,36]. Recombinant inbred line populations generated from multi-parent crosses enable genetic dissection of natural variation and heterosis [51].  In summary, while A. *thaliana* is a selfing species, it retains the ability to outcross and hybridize with related species to varying degrees. The genus *Arabidopsis* provides opportunities to investigate the genetic basis of mating system evolution and interspecific reproductive barriers.  Reference:  [3] Biosynthesis and emission of terpenoid volatiles from *Arabidopsis* flowers, Plant Cell, pmcid: PMC141215  [5] The evolution of selfing in *Arabidopsis* *thaliana*, Science, pmcid: 10.1126/science.1143153  [12] Heterochromatin, small RNA and post-fertilization dysgenesis in allopolyploid and interploid hybrids of *Arabidopsis*, New Phytol, pmcid: PMC3756494  [13] Hybridization alters spontaneous mutation rates in a parent-of-origin-dependent fashion in *Arabidopsis*, Plant Physiol, pmcid: PMC4012600  [14] Dissection of two major components of the post-zygotic hybridization barrier in rice endosperm, Plant J, pmcid: 10.1111/tpj.12333  [15] Generation of self-incompatible *Arabidopsis* *thaliana* by transfer of two S locus genes from A. lyrata, Science, pmcid: 10.1126/science.1072205  [23] The scale of population structure in *Arabidopsis* *thaliana*, PLoS Genet, pmcid: PMC2820523  [25] Inheritance and dominance of self-incompatibility alleles in polyploid *Arabidopsis* lyrata, Heredity (Edinb), pmcid: 10.1038/sj.hdy.6800526  [26] Natural variation in expression of self-incompatibility in *Arabidopsis* *thaliana*: implications for the evolution of selfing, Proc Natl Acad Sci U S A, pmcid: PMC528763  [28] Breakdown of self-incompatibility in the perennial *Arabidopsis* lyrata (Brassicaceae) and its genetic consequences, Evolution, pmid: 16153030  [30] Independent S-locus mutations caused self-fertility in *Arabidopsis* *thaliana*, PLoS Genet, pmcid: PMC2650789  [33] Poorly known relatives of *Arabidopsis* *thaliana*, Trends Plant Sci, pmcid: 10.1016/j.tplants.2006.07.005  [34] Early disruption of maternal-zygotic interaction and activation of defense-like responses in *Arabidopsis* interspecific crosses, Plant Cell, pmcid: PMC3723611  [35] Heterosis of *Arabidopsis* hybrids between C24 and Col is associated with increased photosynthesis capacity, Proc Natl Acad Sci U S A, pmcid: PMC3344962  [36] Assessment of heterosis in two *Arabidopsis* *thaliana* common-reference mapping populations, PLoS One, pmcid: PMC6185836  [38] Maternal epigenetic pathways control parental contributions to *Arabidopsis* early embryogenesis, Cell, pmcid: 10.1016/j.cell.2011.04.014  [42] Darwinian selection on a selfing locus, Science, pmcid: 10.1126/science.1103776  [44] Rice interspecies hybrids show precocious or delayed developmental transitions in the endosperm without change to the rate of syncytial nuclear division, Plant J, pmcid: 10.1111/j.1365-313X.2010.04466.x  [46] Evolutionary dynamics of mating system shifts in *Arabidopsis* lyrata, J Evol Biol, pmcid: 10.1111/j.1420-9101.2010.02073.x  [47] Parent-dependent loss of gene silencing during interspecies hybridization, Curr Biol, pmcid: 10.1016/j.cub.2006.05.045  [51] Analysis of natural allelic variation in *Arabidopsis* using a multiparent recombinant inbred line population, Proc Natl Acad Sci U S A, pmcid: PMC3060268 | 74.89 ± 13.57 |
| GPT3.5 0125 | Query: 1. Does *Arabidopsis* reproduce by self-pollination or hybridization?  ### Response:  *Arabidopsis* primarily reproduces through self-pollination, where pollen is transferred from the anthers of a flower to the stigma of the same flower or another flower on the same plant. While cross-pollination can occur, the predominant mode of reproduction in *Arabidopsis* is self-pollination [1].  References:  [1] Weigel, D. *Arabidopsis*: A key reference genome for understanding flowering time control in plants. Journal of Experimental Botany, 2015, 66(8):2307. 316. DOI: 10.1093/jxb/erv081 | 60.54 ± 13.74 |
| RAG5+0125 | Query: 1. Does *Arabidopsis* reproduce by self-pollination or hybridization?  ### Response:  *Arabidopsis* *thaliana* is primarily known for its ability to self-pollinate; however, it can also undergo outcrossing, albeit at a low frequency, due to intervention or natural factors. Self-pollination occurs when the pollen from the flower fertilizes its own ovules. Yet, certain conditions, such as early stages of flowering where stigmas are not receptive to their own pollen, can influence this process. Additionally, *Arabidopsis* flowers emit volatile compounds, such as monoterpenes and sesquiterpenes, which may attract insect pollinators, thus facilitating outcrossing in wild populations [1][2][3][4]. The complexity of the self-incompatibility locus in other related species further enhances outcrossing mechanisms, although *Arabidopsis* *thaliana* lacks such stringent barriers to self-fertilization [5].  Reference:  [1] Setting up *Arabidopsis* crosses, CSH Protoc, pmcid: 10.1101/pdb.prot4623, pmid: 22485970, url: https://doi.org/10.1101/pdb.prot4623  [2] *Arabidopsis* leucine-rich repeat malectin receptor-like kinases regulate pollen-stigma interactions, Plant Physiol, pmcid: 10.1093/plphys/kiae038, pmid: 38270530, url: https://doi.org/10.1093/plphys/kiae038  [3] Biosynthesis and emission of terpenoid volatiles from *Arabidopsis* flowers, Plant Cell, pmcid: PMC141215, pmid: 12566586, url: http://www.ncbi.nlm.nih.gov/pmc/articles/pmc141215/  [4] Transgenic GFP as a molecular marker for approaches to quantify pollination mechanism and gene flow in *Arabidopsis* *thaliana*, Plant Biol (Stuttg), pmcid: 10.1055/s-2005-837734, pmid: 16025413, url: https://doi.org/10.1055/s-2005-837734  [5] The evolution of selfing in *Arabidopsis* *thaliana*, Science, pmcid: 10.1126/science.1143153, pmid: 17656687, url: https://doi.org/10.1126/science.1143153 | 53.27 ± 20.39 |
| RAG10+0125 | Query: 1. Does *Arabidopsis* reproduce by self-pollination or hybridization?  ### Response:  *Arabidopsis* *thaliana* is generally self-pollinating, meaning it can fertilize itself without external intervention. However, cross-pollination can occur, especially in wild populations where insect-mediated outcrossing is observed, albeit at low frequencies [2][3]. The typical process of self-pollination involves the alignment of stamens and pistils within a flower, facilitating pollen transfer to the stigma [7]. To generate cross-progeny, deliberate crossing between different *Arabidopsis* plants is required, as outlined in detailed protocols [1][4].  Reference:  [1] Setting up *Arabidopsis* crosses, CSH Protoc, pmcid: 10.1101/pdb.prot4623, pmid: 22485970, url: <https://doi.org/10.1101/pdb.prot4623>  [2] *Arabidopsis* leucine-rich repeat malectin receptor-like kinases regulate pollen-stigma interactions, Plant Physiol, pmcid: 10.1093/plphys/kiae038, pmid: 38270530, url: <https://doi.org/10.1093/plphys/kiae038>  [3] Biosynthesis and emission of terpenoid volatiles from *Arabidopsis* flowers, Plant Cell, pmcid: PMC141215, pmid: 12566586, url:  <http://www.ncbi.nlm.nih.gov/pmc/articles/pmc141215/>  [4] Transgenic GFP as a molecular marker for approaches to quantify pollination mechanism and gene flow in *Arabidopsis* *thaliana*, Plant Biol (Stuttg), pmcid: 10.1055/s-2005-837734, pmid: 16025413, url: <https://doi.org/10.1055/s-2005-837734> | 67.10 ± 14.84 |
| RAG15+0125 | Query: 1. Does *Arabidopsis* reproduce by self-pollination or hybridization?  ### Response:  *Arabidopsis* *thaliana*, commonly referred to as *Arabidopsis*, is primarily a self-pollinating plant. However, while it is capable of self-pollination, it can also undergo cross-pollination under certain conditions. This dual capability is facilitated by specific floral structures and genetic mechanisms. *Arabidopsis* flowers are structured in such a way that self-pollination is favored, but they can also be cross-pollinated through various interventions or natural processes.  In *Arabidopsis*, self-pollination occurs when pollen from the same flower fertilizes its own ovules. This is generally the default mode of reproduction for the plant, facilitated by the alignment of the stamen and pistil within the flower [1][7]. Despite this self-pollinating tendency, evidence suggests that *Arabidopsis* can and does engage in outcrossing (cross-pollination) to a limited extent, especially in wild populations where insects may assist in the transfer of pollen [2][3]. This outcrossing is less common but contributes to genetic diversity in the species [5].  The ability of *Arabidopsis* to self-pollinate is supported by its reproductive biology, where the pistil and stamen are well-aligned to promote self-pollination. This alignment allows for effective pollen transfer within the same flower [7]. Nevertheless, outcrossing can occur, particularly when environmental conditions or specific genetic factors come into play [4][8].  In summary, while *Arabidopsis* *thaliana* predominantly self-pollinates due to its floral structure and reproductive mechanisms, it is also capable of cross-pollination, which can contribute to its genetic diversity.  Reference:  [1] Setting up *Arabidopsis* crosses, CSH Protoc, pmcid: 10.1101/pdb.prot4623, pmid: 22485970, url: https://doi.org/10.1101/pdb.prot4623  [2] *Arabidopsis* leucine-rich repeat malectin receptor-like kinases regulate pollen-stigma interactions, Plant Physiol, pmcid: 10.1093/plphys/kiae038, pmid: 38270530, url: <https://doi.org/10.1093/plphys/kiae038>  [3] Biosynthesis and emission of terpenoid volatiles from *Arabidopsis* flowers, Plant Cell, pmcid: PMC141215, pmid: 12566586, url: <http://www.ncbi.nlm.nih.gov/pmc/articles/pmc141215/>  [4] Transgenic GFP as a molecular marker for approaches to quantify pollination mechanism and gene flow in *Arabidopsis* *thaliana*, Plant Biol (Stuttg), pmcid: 10.1055/s-2005-837734, pmid: 16025413, url: <https://doi.org/10.1055/s-2005-837734>  [5] The evolution of selfing in *Arabidopsis* *thaliana*, Science, pmcid: 10.1126/science.1143153, pmid: 17656687, url: <https://doi.org/10.1126/science.1143153> | 66.88 ± 15.16 |
| RAG20+0125 | Query: 1. Does *Arabidopsis* reproduce by self-pollination or hybridization?  ### Response:  *Arabidopsis* *thaliana* is known for its high rate of self-pollination, which is facilitated by the alignment of its reproductive organs. However, it does have mechanisms that allow for outcrossing. *Arabidopsis* flowers can emit volatile compounds, such as terpenes, that may attract pollinators, indicating that while self-pollination is predominant, cross-pollination by insects can occur at a low frequency in wild populations [3][4].  The self-pollination process in *Arabidopsis* is mainly due to the alignment of stamen and pistil, which enables pollen to be transferred within the same flower. This process is effective under normal conditions but can be influenced by external factors like temperature [7][17]. Despite its self-pollinating nature, *Arabidopsis* can produce hybrid progeny if cross-pollination is induced by experimental interventions [1][8].  The plant ability to self-pollinate is a significant evolutionary trait that reduces genetic recombination compared to outcrossing species. Nevertheless, *Arabidopsis* still shows substantial genetic diversity and recombination, suggesting that selfing and recombination can coexist [18].  In summary, while *Arabidopsis* *thaliana* predominantly self-pollinates, it has the potential for cross-pollination under certain conditions. This dual capability contributes to its evolutionary flexibility and genetic diversity.  Reference:  [1] Setting up *Arabidopsis* crosses, CSH Protoc, pmcid: 10.1101/pdb.prot4623, pmid: 22485970, url: https://doi.org/10.1101/pdb.prot4623  [3] Biosynthesis and emission of terpenoid volatiles from *Arabidopsis* flowers, Plant Cell, pmcid: PMC141215, pmid: 12566586, url: http://www.ncbi.nlm.nih.gov/pmc/articles/pmc141215/  [4] Transgenic GFP as a molecular marker for approaches to quantify pollination mechanism and gene flow in *Arabidopsis* *thaliana*, Plant Biol (Stuttg), pmcid: 10.1055/s-2005-837734, pmid: 16025413, url: <https://doi.org/10.1055/s-2005-83773>  [7] EPFL peptide signalling ensures robust self-pollination success under cool temperature stress by aligning the length of the stamen and pistil, Plant Cell Environ, pmcid: 10.1111/pce.14498, pmid: 36419209, url: <https://doi.org/10.1111/pce.14498>  [17] The AtXTH28 gene, a xyloglucan endotransglucosylase/hydrolase, is involved in automatic self-pollination in *Arabidopsis* *thaliana*, Plant Cell Physiol, pmcid: PMC2642609, pmid: 19139039, url: <http://www.ncbi.nlm.nih.gov/pmc/articles/pmc2642609/>  [18] Recombination and gene conversion in a 170-kb genomic region of *Arabidopsis* *thaliana*, Genetics, pmcid: PMC1462186, pmid: 12136029, url: <http://www.ncbi.nlm.nih.gov/pmc/articles/pmc1462186/> | 65.99 ± 15.24 |
| GPT3.5 16k | Query: 1. Does *Arabidopsis* reproduce by self-pollination or hybridization?  ### Response:  *Arabidopsis* *thaliana*, commonly known as thale cress, is a plant species that primarily reproduces through self-pollination. This means that the plant's flowers have both male and female reproductive organs, allowing them to fertilize themselves. However, under certain conditions, *Arabidopsis* can also undergo hybridization by cross-pollination between different individuals of the species. This can occur naturally or be induced in laboratory settings for research purposes. Overall, the default mode of reproduction in *Arabidopsis* is self-pollination.  References:  [1] Weigel, D. Natural Variation in *Arabidopsis*: From Molecular Genetics to Ecological Genomics. Plant Physiology, 2012, 158(1):2-22. DOI: 10.1104/pp.111.189845  [2] Koornneef, M., & Meinke, D. Seeds on the Web: *Arabidopsis* *thaliana*, a Model Genetic Organism. The Plant Cell, 2010, 22(8): 2726-2736. DOI: 10.1105/tpc.110.076398 | 63.42 ± 14.65 |
| RAG5+16k | Query: 1. Does *Arabidopsis* reproduce by self-pollination or hybridization?  ### Response:  Can *Arabidopsis* self-pollinate?  Knowledge: *Arabidopsis* is mostly self-pollinated, but there is evidence suggesting that outcrossing can occur in wild populations [2]. *Arabidopsis* flowers emit terpenes, which are potential olfactory cues for pollinating insects, indicating the possibility of insect-mediated outcrossing [3]. However, the stigma in each *Arabidopsis* flower cannot be pollinated by its own pollen in the early phases of flowering [4]. Despite being capable of self-pollination, *Arabidopsis* has evolved a complex self-incompatibility locus in other related mustard plants to ensure outcrossing [5].  Reference:  [2] *Arabidopsis* leucine-rich repeat malectin receptor-like kinases regulate pollen-stigma interactions, Plant Physiol, pmcid: 10.1093/plphys/kiae038, pmid: 38270530, url: <https://doi.org/10.1093/plphys/kiae038>  [3] Biosynthesis and emission of terpenoid volatiles from *Arabidopsis* flowers, Plant Cell, pmcid: PMC141215, pmid: 12566586, url: <http://www.ncbi.nlm.nih.gov/pmc/articles/pmc141215/>  [4] Transgenic GFP as a molecular marker for approaches to quantify pollination mechanism and gene flow in *Arabidopsis* *thaliana*, Plant Biol (Stuttg), pmcid: 10.1055/s-2005-837734, pmid: 16025413, url: <https://doi.org/10.1055/s-2005-837734>  [5] The evolution of selfing in *Arabidopsis* *thaliana*, Science, pmcid: 10.1126/science.1143153, pmid: 17656687, url: <https://doi.org/10.1126/science.1143153> | 58.29 ± 18.64 |
| RAG10+16k | Query: 1. Does *Arabidopsis* reproduce by self-pollination or hybridization?  ### Response:  *Arabidopsis* *thaliana* is primarily a self-pollinated plant, but it can undergo cross-pollination under specific conditions or with human intervention. In nature, the plant's flowers are adapted to self-pollinate, and its sexual reproduction usually involves the transfer of pollen from the anther to the stigma within the same flower. However, *Arabidopsis* can also experience occasional outcrossing, facilitated by environmental factors or insects that visit the flowers, which produce volatile terpenes attracting pollinators [2][3][4].  The generation of cross-progeny in *Arabidopsis* typically requires manual intervention, as outlined in the protocols for controlled crosses [1]. This intervention ensures that seeds from different parental plants are collected and used to study genetic inheritance or other research objectives.  Reference:  [1] Setting up *Arabidopsis* crosses, CSH Protoc, pmcid: 10.1101/pdb.prot4623, pmid: 22485970, url: <https://doi.org/10.1101/pdb.prot4623>  [2] *Arabidopsis* leucine-rich repeat malectin receptor-like kinases regulate pollen-stigma interactions, Plant Physiol, pmcid: 10.1093/plphys/kiae038, pmid: 38270530, url: <https://doi.org/10.1093/plphys/kiae038>  [3] Biosynthesis and emission of terpenoid volatiles from *Arabidopsis* flowers, Plant Cell, pmcid: PMC141215, pmid: 12566586, url: <http://www.ncbi.nlm.nih.gov/pmc/articles/pmc141215/>  [4] Transgenic GFP as a molecular marker for approaches to quantify pollination mechanism and gene flow in *Arabidopsis* *thaliana*, Plant Biol (Stuttg), pmcid: 10.1055/s-2005-837734, pmid: 16025413, url: <https://doi.org/10.1055/s-2005-837734> | 65.34 ± 14.67 |
| RAG15+16k | Query: 1. Does *Arabidopsis* reproduce by self-pollination or hybridization?  ### Response:  *Arabidopsis* *thaliana* is primarily a self-pollinating plant, which means it usually fertilizes itself without needing pollen from another plant. However, some *Arabidopsis* plants do experience outcrossing, though this occurs at a low frequency. This is facilitated by certain floral traits such as the emission of terpenoid volatiles, which may attract pollinating insects [2][3]. Despite its ability to self-pollinate, *Arabidopsis* also exhibits mechanisms that can prevent self-fertilization under specific conditions, such as when the flower's reproductive organs are not yet fully aligned for self-pollination [4][7]. Additionally, research into the genetic diversity and recombination in *Arabidopsis* shows that, even with a selfing habit, the plant maintains substantial genetic variation [12].  Reference:  [2] *Arabidopsis* leucine-rich repeat malectin receptor-like kinases regulate pollen-stigma interactions, Plant Physiol, pmcid: 10.1093/plphys/kiae038, pmid: 38270530, url: <https://doi.org/10.1093/plphys/kiae038>  [3] Biosynthesis and emission of terpenoid volatiles from *Arabidopsis* flowers, Plant Cell, pmcid: PMC141215, pmid: 12566586, url: <http://www.ncbi.nlm.nih.gov/pmc/articles/pmc141215/>  [4] Transgenic GFP as a molecular marker for approaches to quantify pollination mechanism and gene flow in *Arabidopsis* *thaliana*, Plant Biol (Stuttg), pmcid: 10.1055/s-2005-837734, pmid: 16025413, url: <https://doi.org/10.1055/s-2005-837734>  [7] EPFL peptide signalling ensures robust self-pollination success under cool temperature stress by aligning the length of the stamen and pistil, Plant Cell Environ, pmcid: 10.1111/pce.14498, pmid: 36419209, url: <https://doi.org/10.1111/pce.14498>  [12] Recombination and gene conversion in a 170-kb genomic region of *Arabidopsis* *thaliana*, Genetics, PMCID: PMC1462186, PMID: 12136029, URL: <http://www.ncbi.nlm.nih.gov/pmc/articles/pmc1462186/> | 65.38 ± 14.25 |
| RAG20+16k | Query: 1. Does *Arabidopsis* reproduce by self-pollination or hybridization?  ### Response:  *Arabidopsis* *thaliana* is predominantly self-pollinated, though it is also capable of cross-pollination. In wild populations, low levels of outcrossing can occur, often mediated by insects that are attracted to the plant's floral volatiles [2][3]. The plant sexual reproduction process is mostly self-contained, requiring manual intervention to achieve cross-progeny in experimental settings [1][4]. Key factors such as stamen and pistil alignment are crucial for effective self-pollination [7]. However, barriers to cross-pollination are present, and various genetic and environmental factors can influence the success of hybridization [8][9].  Reference:  [1] Setting up *Arabidopsis* crosses, CSH Protoc, pmcid: 10.1101/pdb.prot4623, pmid: 22485970, url: <https://doi.org/10.1101/pdb.prot4623>  [2] *Arabidopsis* leucine-rich repeat malectin receptor-like kinases regulate pollen-stigma interactions, Plant Physiol, pmcid: 10.1093/plphys/kiae038, pmid: 38270530, url: <https://doi.org/10.1093/plphys/kiae038>  [3] Biosynthesis and emission of terpenoid volatiles from *Arabidopsis* flowers, Plant Cell, pmcid: PMC141215, pmid: 12566586, url: <http://www.ncbi.nlm.nih.gov/pmc/articles/pmc141215/>  [4] Transgenic GFP as a molecular marker for approaches to quantify pollination mechanism and gene flow in *Arabidopsis* *thaliana*, Plant Biol (Stuttg), pmcid: 10.1055/s-2005-837734, pmid: 16025413, url: <https://doi.org/10.1055/s-2005-837734>  [7] EPFL peptide signalling ensures robust self-pollination success under cool temperature stress by aligning the length of the stamen and pistil, Plant Cell Environ, pmcid: 10.1111/pce.14498, pmid: 36419209, url: <https://doi.org/10.1111/pce.14498>  [8] Gamete fertility and ovule number variation in selfed reciprocal F1 hybrid triploid plants are heritable and display epigenetic parent-of-origin effects, New Phytol, pmcid: 10.1111/nph.12147, pmid: 23368793, url: <https://doi.org/10.1111/nph.12147>  [9] Mutational meltdown in selfing *Arabidopsis* lyrata, Evolution, pmcid: 10.1111/j.1558-5646.2012.01818.x, pmid: 23461329, url: <https://doi.org/10.1111/j.1558-5646.2012.01818.x> | 65.93 ± 14.74 |
| GPT4o | Query: 1. Does *Arabidopsis* reproduce by self-pollination or hybridization?  ### Response:  *Arabidopsis* primarily reproduces by self-pollination. The plant has both male (stamens) and female (pistils) reproductive organs, allowing it to fertilize itself. However, it can also be cross-pollinated by environmental factors like wind or insects.  References:  [1] Koornneef M., Meinke D. The development of *Arabidopsis* as a model plant. Plant J. 2010, 61(6):909-921. DOI:10.1111/j.1365-313X.2009.04086.x.  [2] Mitchell-Olds T. *Arabidopsis* *thaliana* and its wild relatives: a model system for ecology and evolution. Trends Ecol Evol. 2001, 16(12):693-700. DOI:10.1016/S0169-5347(01)02291-1. | 66.25 ± 12.68 |
| RAG5+4o | Query: 1. Does *Arabidopsis* reproduce by self-pollination or hybridization?  ### Response:  *Arabidopsis* *thaliana* is predominantly a self-pollinated plant; however, it does possess the capacity for cross-pollination, often facilitated by insects at a low frequency in wild populations[1][3]. The self-pollinating nature of *Arabidopsis* is unique compared to many other members of the mustard family, which rely on a self-incompatibility (S) locus to prevent selfing and ensure outcrossing[5]. Additionally, each flower's stigma in A. *thaliana* is incapable of being pollinated by its own pollen in the initial stages of flowering, allowing for a potential window for cross-pollination[4].  Reference:  [1] Setting up *Arabidopsis* crosses, CSH Protoc, pmcid: 10.1101/pdb.prot4623, pmid: 22485970, url: <https://doi.org/10.1101/pdb.prot4623>  [3] Biosynthesis and emission of terpenoid volatiles from *Arabidopsis* flowers, Plant Cell, pmcid: PMC141215, pmid: 12566586, url: <http://www.ncbi.nlm.nih.gov/pmc/articles/pmc141215/>  [4] Transgenic GFP as a molecular marker for approaches to quantify pollination mechanism and gene flow in *Arabidopsis* *thaliana*, Plant Biol (Stuttg), pmcid: 10.1055/s-2005-837734, pmid: 16025413, url: <https://doi.org/10.1055/s-2005-837734>  [5] The evolution of selfing in *Arabidopsis* *thaliana*, Science, pmcid: 10.1126/science.1143153, pmid: 17656687, url: <https://doi.org/10.1126/science.1143153> | 58.82 ± 17.05 |
| RAG10+4o | Query: 1. Does *Arabidopsis* reproduce by self-pollination or hybridization?  ### Response:  *Arabidopsis* *thaliana* is widely recognized as a predominantly self-pollinating plant. This self-pollination occurs due to the typical alignment of stamen and pistil lengths within a single flower, facilitating the transfer of pollen from the stamen to the stigma [7]. Although self-pollination is the norm, there is evidence that suggests a low frequency of insect-mediated outcrossing in wild populations, which is facilitated by the emission of olfactory cues such as monoterpenes and sesquiterpenes from *Arabidopsis* flowers [3]. Furthermore, while *Arabidopsis* *thaliana* has evolved to self-pollinate, its close relatives in the mustard family exhibit outcrossing behaviors largely due to a complex self-incompatibility locus which prevents self-fertilization [5].  Reference:  [1] Setting up *Arabidopsis* crosses, CSH Protoc, pmcid: 10.1101/pdb.prot4623, pmid: 22485970, url: https://doi.org/10.1101/pdb.prot4623  [3] Biosynthesis and emission of terpenoid volatiles from *Arabidopsis* flowers, Plant Cell, pmcid: PMC141215, pmid: 12566586, url: http://www.ncbi.nlm.nih.gov/pmc/articles/pmc141215/  [5] The evolution of selfing in *Arabidopsis* *thaliana*, Science, pmcid: 10.1126/science.1143153, pmid: 17656687, url: https://doi.org/10.1126/science.1143153  [7] EPFL peptide signalling ensures robust self-pollination success under cool temperature stress by aligning the length of the stamen and pistil, Plant Cell Environ, pmcid: 10.1111/pce.14498, pmid: 36419209, url: https://doi.org/10.1111/pce.14498 | 63.38 ± 14.05 |
| RAG15+4o | Query: 1. Does *Arabidopsis* reproduce by self-pollination or hybridization?  ### Response:  *Arabidopsis* *thaliana* is predominantly a self-pollinating plant. This self-pollination results when the stamen and pistil lengths are aligned within a single flower, allowing the pollen from the stamen to be transferred directly to the stigma [7]. However, it is also worth noting that insect-mediated outcrossing can occur at a low frequency in wild populations due to the emission of olfactory cues like monoterpenes and sesquiterpenes that attract pollinating insects [3].  Despite the high rate of self-pollination, *Arabidopsis* *thaliana* exhibits substantial genetic recombination, which contributes to its genetic diversity [12]. For experimental purposes, crossing *Arabidopsis* plants usually requires intervention by researchers due to its natural tendency to self-pollinate [1][9].  Reference:  [1] Setting up *Arabidopsis* crosses, CSH Protoc, PMCID: 10.1101/pdb.prot4623, PMID: 22485970, URL: <https://doi.org/10.1101/pdb.prot4623>  [3] Biosynthesis and emission of terpenoid volatiles from *Arabidopsis* flowers, Plant Cell, PMCID: PMC141215, PMID: 12566586, URL: <http://www.ncbi.nlm.nih.gov/pmc/articles/pmc141215/>  [7] EPFL peptide signalling ensures robust self-pollination success under cool temperature stress by aligning the length of the stamen and pistil, Plant Cell Environ, PMCID: 10.1111/pce.14498, PMID: 36419209, URL: <https://doi.org/10.1111/pce.14498>  [9] A DNA methyltransferase homolog with a chromodomain exists in multiple polymorphic forms in *Arabidopsis*, Genetics, PMCID: PMC1460135, PMID: 9584105, URL: <http://www.ncbi.nlm.nih.gov/pmc/articles/pmc1460135/>  [12] Recombination and gene conversion in a 170-kb genomic region of *Arabidopsis* *thaliana*, Genetics, PMCID: PMC1462186, PMID: 12136029, URL: <http://www.ncbi.nlm.nih.gov/pmc/articles/pmc1462186/> | 63.54 ± 16.44 |
| RAG20+4o | Query: 1. Does *Arabidopsis* reproduce by self-pollination or hybridization?  ### Response:  *Arabidopsis* *thaliana* primarily self-pollinates, facilitating its own fertilization and seed production without the need for external pollinators. Its ability to self-pollinate is mechanically supported by the alignment of stamen and pistil lengths within the same flower, ensuring that pollen from the anthers can reach the stigma of the same flower[7]. However, while it is mostly self-pollinated, insect-mediated outcrossing can occur at a low frequency in natural populations, indicating that *Arabidopsis* can also undergo occasional cross-pollination induced by external factors[3], [11]. Due to its natural propensity for selfing, controlled cross-pollination experiments require manual interventions by researchers[1].  Reference:  [1] Setting up *Arabidopsis* crosses, CSH Protoc, pmcid: 10.1101/pdb.prot4623, pmid: 22485970, url: <https://doi.org/10.1101/pdb.prot4623>  [3] Biosynthesis and emission of terpenoid volatiles from *Arabidopsis* flowers, Plant Cell, pmcid: PMC141215, pmid: 12566586, url: <http://www.ncbi.nlm.nih.gov/pmc/articles/pmc141215/>  [7] EPFL peptide signalling ensures robust self-pollination success under cool temperature stress by aligning the length of the stamen and pistil, Plant Cell Environ, pmcid: 10.1111/pce.14498, pmid: 36419209, url: <https://doi.org/10.1111/pce.14498>  [11] A DNA methyltransferase homolog with a chromodomain exists in multiple polymorphic forms in *Arabidopsis*, Genetics, pmcid: PMC1460135, pmid: 9584105, url: <http://www.ncbi.nlm.nih.gov/pmc/articles/pmc1460135/> | 63.56 ± 15.00 |
| RAG – Tan + 4o | Query: 1. Does *Arabidopsis* reproduce by self-pollination or hybridization?  ### Response:  *Arabidopsis* *thaliana* is predominantly self-pollinated, meaning that it typically fertilizes its own flowers. This self-pollination mechanism in A. *thaliana* is facilitated by the alignment of stamen and pistil lengths within a single flower, ensuring that pollen from the stamen directly contacts the stigma to initiate fertilization [7]. However, A. *thaliana* can also outcross under certain circumstances, although this occurs much less frequently and often involves intervention by researchers or insect-mediated processes in wild populations [1], [3].  In terms of its reproductive biology, A. *thaliana* has lost the self-incompatibility (SI) system that prevents self-fertilization in many other plant species in the mustard family. This is due to the inactivation of specific genes at the S-locus, which are responsible for identifying and rejecting self-pollen [5]. Consequently, A. *thaliana* can self-fertilize efficiently without the genetic barriers that typically enforce outcrossing in related species[15].  In summary, while *Arabidopsis* *thaliana* primarily relies on self-pollination, it can exhibit outcrossing under certain conditions, and the loss of self-incompatibility facilitates its selfing nature.  Reference:  [1] Setting up *Arabidopsis* crosses, CSH Protoc, pmcid: 10.1101/pdb.prot4623, pmid: 22485970, url: <https://doi.org/10.1101/pdb.prot4623>  [3] Biosynthesis and emission of terpenoid volatiles from *Arabidopsis* flowers, Plant Cell, pmcid: PMC141215, pmid: 12566586, url: <http://www.ncbi.nlm.nih.gov/pmc/articles/pmc141215/>  [5] The evolution of selfing in *Arabidopsis* *thaliana*, Science, pmcid: 10.1126/science.1143153, pmid: 17656687, url: <https://doi.org/10.1126/science.1143153>  [7] EPFL peptide signalling ensures robust self-pollination success under cool temperature stress by aligning the length of the stamen and pistil, Plant Cell Environ, pmcid: 10.1111/pce.14498, pmid: 36419209, url: <https://doi.org/10.1111/pce.14498>  [15] Generation of self-incompatible *Arabidopsis* *thaliana* by transfer of two S locus genes from A. lyrata, Science, pmcid: 10.1126/science.1072205, pmid: 12114625, url: https://doi.org/10.1126/science.1072205 10.1126/science.1072205, pmid: 12114625, url: https://doi.org/10.1126/science.1072205 | 65.45 ± 15.63 |
| Inference problem：RAG-Tan + Opus | Query: 1. If the expression of the FLC gene is completely suppressed while the expression of the FT gene significantly increases, what effect might this have on the flowering time of *Arabidopsis*? Consider the role of vernalization.  ### Response:  Based on the information provided, if FLC expression is completely suppressed while FT expression significantly increases, this would likely result in earlier flowering time in *Arabidopsis*, even in the absence of vernalization.  FLC acts as a potent floral repressor in *Arabidopsis* [1,6,13]. It delays flowering by suppressing the expression of floral promoters like FT and SOC1 [4,12]. Vernalization normally leads to epigenetic silencing of FLC, which allows flowering to proceed [9,14,17].  However, even if FLC function is completely lost, this does not entirely eliminate the vernalization response, suggesting vernalization can also promote flowering through FLC-independent mechanisms [25]. Nonetheless, FLC is considered the central regulator of the vernalization response [13].  Conversely, FT acts as a key floral promoter. Overexpression of FT leads to early flowering [4,26]. FT is normally repressed by FLC, but vernalization relieves this repression by silencing FLC [12,20].  Therefore, if FLC is fully suppressed while FT is highly expressed, this mimics the vernalized state where the floral repressor is inactivated and the floral promoter is induced. This would bypass the normal requirement for vernalization and lead to early flowering, since the two major antagonistic regulators of flowering are artificially set to a state that promotes the transition to flowering.  In summary, complete suppression of FLC combined with high induction of FT would likely cause early flowering in *Arabidopsis* by mimicking the effects of vernalization on these two key flowering time genes, even in the absence of an actual cold treatment. The plant would be in a derepressed state without the floral inhibitor and with strong activation of the floral promoter.  Reference:  [1] FLOWERING LOCUS C encodes a novel MADS domain protein that acts as a repressor of flowering, Plant Cell, pmcid: PMC144226  [4] Integration of flowering signals in winter-annual *Arabidopsis*, Plant Physiol, pmcid: PMC548846  [6] Regulation of flowering in *Arabidopsis* by an FLC homologue, Plant Physiol, pmcid: PMC102287  [9] Vernalization requires epigenetic silencing of FLC by histone methylation, Nature, pmcid: 10.1038/nature02269  [12] The transcription factor FLC confers a flowering response to vernalization by repressing meristem competence and systemic signaling in *Arabidopsis*, Genes Dev, pmcid: PMC1472290  [13] The molecular basis of vernalization: the central role of FLOWERING LOCUS C (FLC), Proc Natl Acad Sci U S A, pmcid: PMC16312  [14] A cluster of *Arabidopsis* genes with a coordinate response to an environmental stimulus, Curr Biol, pmcid: 10.1016/j.cub.2004.04.045  [17] Vernalization: a model for investigating epigenetics and eukaryotic gene regulation in plants, Biochim Biophys Acta, pmcid: 10.1016/j.bbaexp.2007.02.003  [20] Differential regulation of FLOWERING LOCUS C expression by vernalization in cabbage and *Arabidopsis*, Plant Physiol, pmcid: PMC1065404  [25] Loss of FLOWERING LOCUS C activity eliminates the late-flowering phenotype of FRIGIDA and autonomous pathway mutations but not responsiveness to vernalization, Plant Cell, pmcid: PMC135534  [26] The FT/TFL1 gene family in grapevine, Plant Mol Biol, pmcid: 10.1007/s11103-006-9113-z | 86.17±6.006 |
| Inference problem：OpenAI o1 | 1. If the expression of the FLC gene is completely suppressed while the expression of the FT gene significantly increases, what effect might this have on the flowering time of *Arabidopsis*? Consider the role of vernalization.  ### Response:  Suppressing the expression of the **FLC** (FLOWERING LOCUS C) gene while significantly increasing the expression of the **FT** (FLOWERING LOCUS T) gene would lead to earlier flowering in **Arabidopsis* *thaliana**.  **FLC** is a key floral repressor that delays flowering by inhibiting the expression of flowering-promoting genes like **FT** and **SOC1** (SUPPRESSOR OF OVEREXPRESSION OF CONSTANS 1) [1][2]. It encodes a MADS-box transcription factor that binds to the promoters of these genes, preventing their activation. In **Arabidopsis**, high levels of **FLC** expression keep the plant in a vegetative state, delaying the transition to flowering.  **Vernalization** is the process by which prolonged exposure to cold temperatures leads to the epigenetic suppression of **FLC** expression [3]. During vernalization, histone modifications at the **FLC** locus result in a stable, heritable suppression of **FLC**, lifting its repressive effect on **FT** and allowing the plant to flower when temperatures rise [4]. This adaptation ensures that flowering occurs under favorable conditions after winter.  By completely suppressing **FLC** expression artificially (e.g., through genetic manipulation), the plant bypasses the need for vernalization to initiate flowering. Without **FLC** to repress it, **FT** expression can increase. **FT** acts as a crucial promoter of flowering; it encodes a mobile protein known as **florigen** that moves from the leaves to the shoot apical meristem to trigger the flowering process [5][6].  An increase in **FT** expression amplifies the flowering signal. Elevated levels of **FT** can promote the expression of floral identity genes more robustly, accelerating the transition from vegetative growth to flowering even further [7]. This combination of removing a repressor (**FLC**) and enhancing an activator (**FT**) creates a strong promotive effect on flowering time.  In practical terms, the plant would flower earlier than it normally would under natural conditions. This early flowering occurs regardless of exposure to cold temperatures because the genetic requirement for vernalization (suppression of **FLC**) has been met through direct suppression. The significant increase in **FT** ensures that the flowering signals are strong and effective in inducing the development of flowers.  In summary, complete suppression of **FLC** removes inhibitory control over flowering-promoting genes, and elevated **FT** expression enhances the flowering signals. Together, these changes lead to precocious flowering in **Arabidopsis**, bypassing the natural vernalization requirement and potentially altering the plant's normal developmental timing.  **Reference:**  [1] Michaels SD, Amasino RM. FLOWERING LOCUS C encodes a novel MADS domain protein that acts as a repressor of flowering. *Plant Cell*. 1999 May;11(5):949-56. PMID: 10330478.  [2] Searle I, Hepworth SR, Grandi V, Kouzarides T, Dean C. The transcriptional repressor vernalization1 (VRN1) controls developmental timing in **Arabidopsis** by repressing FLC through chromatin modification. *Genes Dev*. 2006 Jan 15;20(2):166-70. PMID: 16418484.  [3] Amasino R. Vernalization and flowering time. *Curr Opin Biotechnol*. 2005 Apr;16(2):154-8. PMID: 15831384.  [4] Sung S, Amasino RM. Vernalization in **Arabidopsis* *thaliana** is mediated by the PHD finger protein VIN3. *Nature*. 2004 Jan 29;427(6970):159-64. PMID: 14712277.  [5] Corbesier L et al. FT protein movement contributes to long-distance signaling in floral induction of **Arabidopsis**. *Science*. 2007 May 4;316(5827):1030-3. PMID: 17446353.  [6] Turck F, Fornara F, Coupland G. Regulation and identity of florigen: FLOWERING LOCUS T moves center stage. *Annu Rev Plant Biol*. 2008;59:573-94. PMID: 18257709.  [7] Kardailsky I et al. Activation tagging of the floral inducer FT. *Science*. 1999 Dec 3;286(5446):1962-5. PMID: 10583959. | 88.17±4.684 |
| Inference problem：RAG-Tan + o1 | Query: 1. If the expression of the FLC gene is completely suppressed while the expression of the FT gene significantly increases, what effect might this have on the flowering time of *Arabidopsis*? Consider the role of vernalization.  ### Response:  Suppressing the expression of the FLC gene in *Arabidopsis* would likely lead to an acceleration of flowering time. FLC (FLOWERING LOCUS C) functions as a major repressor of flowering by inhibiting the expression of genes that promote flowering, including FT (FLOWERING LOCUS T) [1][13][22]. When FLC expression is completely suppressed, this repression is lifted, allowing FT expression to increase significantly [20][40].  FT is a key floral integrator gene that promotes the transition from vegetative growth to flowering. An increase in FT expression enhances the production of the florigen signal, which induces flowering in the shoot apical meristem [26][40]. Therefore, the significant increase in FT expression, in the absence of FLC-mediated repression, would result in earlier flowering than would normally occur.  Vernalization, the process by which prolonged exposure to cold leads to flowering, operates primarily through the epigenetic repression of FLC [13][17][22]. In winter-annual ecotypes of *Arabidopsis*, high levels of FLC expression inhibit flowering until the plant experiences prolonged cold, which then suppresses FLC expression and permits flowering [13][17]. If FLC is already completely suppressed without the need for cold exposure, the requirement for vernalization is effectively bypassed [25]. This means that the plant would flower earlier, even without experiencing the prolonged cold periods typically necessary to induce flowering in winter-annual types.  In summary, the complete suppression of FLC expression combined with a significant increase in FT expression would lead to early flowering in *Arabidopsis*. This effect mimics the outcome of vernalization by removing the repression exerted by FLC on flowering-promoting genes, thereby accelerating the flowering process without the need for prolonged cold exposure.  **References:**  [1] FLOWERING LOCUS C encodes a novel MADS domain protein that acts as a repressor of flowering, *Plant Cell*, PMCID: PMC144226, PMID: 10330478, URL: http://www.ncbi.nlm.nih.gov/pmc/articles/pmc144226/  [13] The molecular basis of vernalization: the central role of FLOWERING LOCUS C (FLC), *Proc Natl Acad Sci U S A*, PMCID: PMC16312, PMID: 10716723, URL: http://www.ncbi.nlm.nih.gov/pmc/articles/pmc16312/  [17] Vernalization: a model for investigating epigenetics and eukaryotic gene regulation in plants, *Biochim Biophys Acta*, DOI: 10.1016/j.bbaexp.2007.02.003, PMID: 17383745, URL: https://doi.org/10.1016/j.bbaexp.2007.02.003  [20] Differential regulation of FLOWERING LOCUS C expression by vernalization in cabbage and *Arabidopsis*, *Plant Physiol*, PMCID: PMC1065404, PMID: 15734903, URL: http://www.ncbi.nlm.nih.gov/pmc/articles/pmc1065404/  [22] FLC or not FLC: the other side of vernalization, *J Exp Bot*, DOI: 10.1093/jxb/ern070, PMID: 18390846, URL: https://doi.org/10.1093/jxb/ern070  [25] Loss of FLOWERING LOCUS C activity eliminates the late-flowering phenotype of FRIGIDA and autonomous pathway mutations but not responsiveness to vernalization, *Plant Cell*, PMCID: PMC135534, PMID: 11283346, URL: http://www.ncbi.nlm.nih.gov/pmc/articles/pmc135534/  [26] The FT/TFL1 gene family in grapevine, *Plant Mol Biol*, DOI: 10.1007/s11103-006-9113-z, PMID: 17160562, URL: https://doi.org/10.1007/s11103-006-9113-z  [40] Analysis of the *Arabidopsis* MADS AFFECTING FLOWERING gene family: MAF2 prevents vernalization by short periods of cold, *Plant Cell*, PMCID: PMC153723, PMID: 12724541, URL: http://www.ncbi.nlm.nih.gov/pmc/articles/pmc153723/ | 86.57±5.315 |

Data are presented as mean ± SD, n=110.

Note：The highlighted information is the hallucinatory information, the hallucinatory information is not fully labeled.

Extended Table 2. Extreme questions

| Fractional range | ≥ 80. 61 | ≤ 48.97 |
| --- | --- | --- |
| Questions | 5. How do ABA and SA affect the level of cellular NPR1 protein? What changes will occur in NPR1 protein levels and target gene expression after continuous SA and ABA treatment?  9. In Arabidopsis, what effects do the EDS1 complex and PAD4 have on MYC2? What effects will the effects on MYC2 have on SA?  11. What role does N-hydroxyisopiperidinic acid (N-OH-Pip) play in Arabidopsis? What effects will exogenous application of N-OH-Pip have on Arabidopsis?  20. What role does ZmEREB20 play in Arabidopsis? What traits do ZmEREB20 overexpression lines show under stress conditions?  41. The mechanism of Arabidopsis CAMTA123 regulating salicylic acid  42. Taking Arabidopsis as an example, explain the process of FC detoxification and recycling  43. How does Arabidopsis camalexin respond to pathogen infection  44. Arabidopsis ACOS5 is mainly expressed in flowers. What is its specific function? How does it function?  46. I would like to know about the Arabidopsis AtGA3ox2 gene  47. I would like to know about Arabidopsis MYB15  49. I would like to know about the Arabidopsis ADC2 gene  51. How does the SPL transcription factor regulated by miR156 define the endogenous flowering pathway of Arabidopsis  52. Please tell me the role of Arabidopsis AP2/EREBP-type transcription repressors in abscisic acid and drought stress responses  53. Please tell me the effect of microRNA160 on seed germination  54. Is there a correlation between the functions of MYC2 and SPA1 in the development of Arabidopsis light seedlings  55. Arabidopsis RING-H2 protein RHA2a in ABA Biological functions in signal transduction  56. Nitrate partially relieves dormancy of Arabidopsis seeds by reducing abscisic acid (ABA) levels. Which gene is involved?  57. Please tell me the role of CpMYB10 in Arabidopsis stress resistance  59. Please tell me how MYB101 and MYB33 transcription factors respond to ABA during seed germination  60. Please tell me what role MYB96 transcription factor plays in the drought stress response process of Arabidopsis  85. In the study of Arabidopsis gene expression levels, are the spatiotemporal transcriptome data completely reliable?  91. Does Arabidopsis reproduce by self-pollination or hybridization?  92. Why was Arabidopsis selected as a model plant for plant genetic transformation?  93. What is the distribution range of Arabidopsis?  94. What is the significance of studying Arabidopsis?  95. What is the significance of studying Arabidopsis for rice genetics?  96. What is the family of Arabidopsis?  97. How many pairs of chromosomes does Arabidopsis have?  98. What is the fruit shape of Arabidopsis?  99. What is the medicinal value of Arabidopsis?  100. How many base pairs are there in the Arabidopsis genome? | 2. What does the plant toxin thaxtomin A (TXA) in Arabidopsis rely on to stimulate the expression of FMO1 protein?  3. What inhibits the NRM inhibitory activity in Arabidopsis?  4. What will happen if CBP60g is overexpressed in Arabidopsis? What will happen if the CBP60g overexpression line is overexpressed?  10. What stress response genes does CAMTA1 regulate? What can be regulated to regulate drought recovery?  17. What role does MHP1 play in Arabidopsis? What is its relationship with Mpo1?  18. What regulates the accumulation of flavonols in Arabidopsis? What role do COP1 and MYB12 play in the biosynthesis of flavonols in Arabidopsis?  19. What role does HAP5A play in Arabidopsis? How does it work? What effects does the lack of HAP5A have on Arabidopsis?  21. What genes inhibit the accumulation of flavonoids in Arabidopsis? With the help of synthetic biology and genetic engineering methods and related mechanisms and synthesis pathways in Arabidopsis, try to explain the method of synthesizing flavonoids in large quantities in plants?  22. What genes or enzymes are related to the burst of reactive oxygen in Arabidopsis? What phenotypes will the burst of reactive oxygen cause in Arabidopsis? What treatment conditions of Arabidopsis are ROS also related to?  28. What phenotypes will appear under the condition of nitrogen deficiency in Arabidopsis? Under nitrogen deficiency, what molecular mechanism causes these phenotypes in plants?  29. Can Arabidopsis be improved through genetic engineering methods to become a plant that prevents sand and fixes soil?  30. Will Arabidopsis have lodging phenomenon? If it does, what substance in the body is reduced? And what internal mechanism is it related to? What gene can be overexpressed to alleviate or resist lodging of this phenotype?  35. Will Arabidopsis experience a transition in the nutritional period? What is the process of transition in the nutritional period of Arabidopsis? Which genes are involved? How do these genes work to cause Arabidopsis to undergo a transition in the nutritional period? Which nutritional organs of Arabidopsis are related to this process? What aspects can be used to study the transition in the nutritional period of Arabidopsis?  38. What developmental parts and genes of Arabidopsis are related to DNA damage in Arabidopsis seed germination?  40. Can overexpressing FLM achieve flowering inhibition in Arabidopsis?  58. Please tell me the function of HOS10 in Arabidopsis cold resistance  64. What are the methods for constructing mutant libraries using Arabidopsis?  71. Functions of NADK1?  72. What are the signal transduction pathways involved in NADK1?  73. Which transcription factors are involved in the regulation of ICL expression?  75. What important metabolic pathways does succinate participate in?  76. What are the key genes co-regulate light and retrograde signals?  80. The differences between the effects of sucrose and glucose in the early morphogenesis of Arabidopsis? |

Extended Table 3. Phenotypic - gene task effect comparison

| Model | Phenotypic - Current status of gene research |
| --- | --- |
| RAG5 + Llama3 8B | Query: 1. How does *Arabidopsis* regulate the balance between its own development and drought stress response?  ### Response:  *Arabidopsis* regulates the balance between its own development and drought stress response through several mechanisms:  1. ABA-mediated signaling: *Arabidopsis* utilizes abscisic acid (ABA) as a key hormone in drought stress signaling. ABA activates large-scale alterations in gene expression to improve survival under water-deficit conditions [1][3].  2. Transcription factor regulation: Homeodomain-leucine zipper class I genes, specifically ATHB7 and ATHB12, are strongly induced by drought stress and ABA. These transcription factors modulate ABA signaling by regulating protein phosphatase 2C and ABA receptor gene activities [3].  3. Protein kinase cooperation: Two closely related subclass II SnRK2 protein kinases work together to regulate drought-inducible gene expression, contributing to the plant's adaptive response [4].  4. Stomatal regulation: Genes like ZmHK9 mediate drought tolerance through the regulation of stomatal development, allowing the plant to control water loss [5].  5. Negative regulation: Some genes, such as MSI1, are required for negative regulation of the drought stress response, potentially preventing an overreaction that could hinder normal development [2].  These mechanisms allow *Arabidopsis* to respond effectively to drought stress while maintaining essential developmental processes.  Reference:  [1] Mutations in ABO1/ELO2, a subunit of holo-Elongator, increase abscisic acid sensitivity and drought tolerance in *Arabidopsis* *thaliana*, Mol Cell Biol, PMCID: PMC159285  [2] *Arabidopsis* MSI1 is required for negative regulation of the response to drought stress, Mol Plant, PMID: 19825648  [3] The homeodomain-leucine zipper (HD-Zip) class I transcription factors ATHB7 and ATHB12 modulate abscisic acid signalling by regulating protein phosphatase 2C and abscisic acid receptor gene activities, Plant Mol Biol, PMID: 22968620  [4] Two closely related subclass II SnRK2 protein kinases cooperatively regulate drought-inducible gene expression, Plant Cell Physiol, PMID: 20375108  [5] A novel histidine kinase gene, ZmHK9, mediate drought tolerance through the regulation of stomatal development in *Arabidopsis*, Gene, PMID: 22525037 |
| RAG10 + 0125 | Query: 1. How does *Arabidopsis* regulate the balance between its own development and drought stress response?  ### Response:  In *Arabidopsis* *thaliana*, abscisic acid (ABA) plays a crucial role in the plant's response to drought stress. ABA signaling is central to modulating drought responses through various mechanisms:  1. **Transcriptional Regulation**: ABA influences the expression of genes involved in drought tolerance. Specifically, the homeodomain-leucine zipper (HD-Zip) class I transcription factors ATHB7 and ATHB12 are regulated by ABA and contribute to the modulation of drought responses by impacting protein phosphatase  2C and ABA receptor gene activities [3].  2. **Protein Kinase Activity**: SnRK2 protein kinases, particularly subclass II kinases, are involved in regulating drought-inducible gene expression. These kinases cooperate to enhance the plant's ability to handle drought conditions [4].  3. **Gene Expression and Stress Tolerance**: Overexpression of certain genes like AtBBD1 has been shown to enhance drought tolerance by increasing the expression of ABA-responsive genes and accumulating proline, which is a protective compound against stress [8]. AtBBD1 also interacts with other regulatory proteins to bolster drought responses [8].  4. **Drought Stress and Glucose Signaling**: Studies also show that glucose signaling, along with ABA signaling, plays a role in stress tolerance and drought response mechanisms [7].  Overall, ABA-mediated pathways are integral to enhancing drought tolerance in *Arabidopsis* through complex networks of gene regulation and protein interactions.  Reference:  [3] The homeodomain-leucine zipper (HD-Zip) class I transcription factors ATHB7 and ATHB12 modulate abscisic acid signalling by regulating protein phosphatase 2C and abscisic acid receptor gene activities, Plant Mol Biol, pmcid: 10.1007/s11103-012-9956-4, pmid: 22968620, url: <https://doi.org/10.1007/s11103-012-9956-4>  [4] Two closely related subclass II SnRK2 protein kinases cooperatively regulate drought-inducible gene expression, Plant Cell Physiol, pmcid: 10.1093/pcp/pcq041, pmid: 20375108, url: <https://doi.org/10.1093/pcp/pcq041>  [7] Stress tolerance and glucose insensitive phenotypes in *Arabidopsis* overexpressing the CpMYB10 transcription factor gene, Plant Physiol, pmcid: PMC429382, pmid: 15122027, url: <http://www.ncbi.nlm.nih.gov/pmc/articles/pmc429382/>  [8] Overexpression of AtBBD1, *Arabidopsis* Bifunctional Nuclease, Confers Drought Tolerance by Enhancing the Expression of Regulatory Genes in ABA-Mediated  Drought Stress Signaling, Int J Mol Sci, pmcid: PMC8001636, pmid: 33805821, url: <http://www.ncbi.nlm.nih.gov/pmc/articles/pmc8001636/> |
| Claude Opus | Query: 1. How does *Arabidopsis* regulate the balance between its own development and drought stress response?  ### Response:  *Arabidopsis* regulates the balance between its development and drought stress response through several key mechanisms:  1. Abscisic acid (ABA) signaling: ABA accumulates during drought stress and triggers stomatal closure to reduce water loss. It also inhibits growth and promotes stress-responsive gene expression. ABA-insensitive mutants show reduced drought tolerance but enhanced growth under stress [1].  2. Transcription factors: Drought-responsive transcription factors like DREB2A and AREB1 activate stress-responsive genes involved in osmotic adjustment, detoxification, and growth regulation. Overexpression of these factors improves drought tolerance but often stunts growth [2].  3. Gibberellin (GA) signaling: Drought stress reduces bioactive GA levels, leading to DELLA protein accumulation. DELLAs restrict growth and enhance stress tolerance by modulating transcription factors and ABA signaling. GA application reverses these effects [3].  4. Crosstalk between hormones: Interactions between ABA, GA, auxin, cytokinin, and other hormones fine-tune the balance between stress adaptation and growth. For example, ABA inhibits GA biosynthesis, while cytokinin enhances ABA degradation [4].  5. Epigenetic regulation: Drought-induced chromatin modifications and small RNAs modulate gene expression, allowing long-term adaptation to stress. These changes can be inherited across generations, potentially improving offspring fitness under stress [5].  In summary, *Arabidopsis* employs a complex network of hormonal, transcriptional, and epigenetic mechanisms to dynamically adjust its development and stress response based on environmental conditions.  References:  [1] Finkelstein, R. Abscisic Acid Synthesis and Response. The *Arabidopsis* Book, 2013, 11:e0166. <https://doi.org/10.1199/tab.0166>  [2] Yoshida, T. et al. AREB1, AREB2, and ABF3 are master transcription factors that cooperatively regulate ABRE-dependent ABA signaling involved in drought stress tolerance and require ABA for full activation. The Plant Journal, 2010, 61(4):672-685. <https://doi.org/10.1111/j.1365-313X.2009.04092.x>  [3] Colebrook, E.H. et al. The role of gibberellin signalling in plant responses to abiotic stress. Journal of Experimental Biology, 2014, 217(1):67-75. <https://doi.org/10.1242/jeb.089938>  [4] Huang, D. et al. The relationship of drought-related gene expression in *Arabidopsis* *thaliana* to hormonal and environmental factors. Journal of Experimental Botany, 2008, 59(11):2991-3007. <https://doi.org/10.1093/jxb/ern155>  [5] Chinnusamy, V. & Zhu, J.K. Epigenetic regulation of stress responses in plants. Current Opinion in Plant Biology, 2009, 12(2):133-139. <https://doi.org/10.1016/j.pbi.2008.12.006> |

Extended Table 4. Gene function task effect comparison

| Model | Current status of gene function research |
| --- | --- |
| RAG5 + Llama3 8B | Query: 1. What effects do CAMTA proteins have in *Arabidopsis* under low temperature, warm conditions, and high temperature conditions?  ### Response:  CAMTA proteins in *Arabidopsis* have different effects under various temperature conditions:  1. Low temperature (4 ℃):  - CAMTA3 rapidly induces CBF1 and CBF2 genes [3].  - CAMTA1, CAMTA2, and CAMTA3 collectively activate many cold-responsive genes within 24 hours, increasing plant freezing tolerance [3].  - Prolonged exposure (>1 week) leads to increased SA levels due to reduced CAMTA-mediated repression [1][3].  2. Warm conditions (22 ℃):  - CAMTA1, CAMTA2, and CAMTA3 work together to repress SA biosynthesis and SA-mediated immunity genes [1][3].  3. High temperature:  The provided information does not specifically mention effects at high temperatures.  It's worth noting that CAMTA3's regulation of SA-related genes is mediated through its DNA binding domain, independent of its calmodulin-binding domains [2].  Reference:  [1] CAMTA-Mediated Regulation of Salicylic Acid Immunity Pathway Genes in *Arabidopsis* Exposed to Low Temperature and Pathogen Infection, Plant Cell, PMCID: PMC5774559  [2] Temperature modulation of CAMTA3 gene induction activity is mediated through the DNA binding domain, Plant J, PMCID: PMC9826522  [3] Roles of CAMTA transcription factors and salicylic acid in configuring the low-temperature transcriptome and freezing tolerance of *Arabidopsis*, Plant J, PMID: 23581962 |
| RAG10 + 0125 | Query: 1. What effects do CAMTA proteins have in *Arabidopsis* under low temperature, warm conditions, and high temperature conditions?  ### Response:  The *Arabidopsis* CAMTA transcription factors play important roles in regulating salicylic acid (SA) biosynthesis and SA-mediated immunity in response to temperature changes. At warm temperatures (22℃), CAMTA factors, particularly CAMTA1, CAMTA2, and CAMTA3, repress the expression of genes involved in SA biosynthesis and SA-mediated immunity [1, 3]. However, when plants are exposed to low temperatures (4℃) for more than a week, this repression is overcome, leading to increased SA levels [1, 3].  CAMTA3 is also involved in the rapid cold induction of CBF genes, which contribute to freezing tolerance in plants [2]. The temperature modulation of CAMTA3 gene induction activity is mediated through its DNA binding domain and occurs independently of the C-terminal calmodulin-binding domains [2]. Domain-swapping experiments suggest that the repression activity of the N-terminal region of CAMTA3 is suppressed by the action of the IQ and calmodulin-binding domains in response to low-temperature signals [1, 2].  While SA biosynthesis at low temperatures does not directly contribute to freezing tolerance, it plays a major role in configuring the low-temperature transcriptome of *Arabidopsis* [3]. The increased SA levels at low temperatures are likely due to post-transcriptional regulation of CAMTA factors, as their transcript levels do not decrease at low temperatures [3].  References:  [1] CAMTA-Mediated Regulation of Salicylic Acid Immunity Pathway Genes in *Arabidopsis* Exposed to Low Temperature and Pathogen Infection, Plant Cell, PMCID: PMC5774559, PMID: 28982964  [2] Temperature modulation of CAMTA3 gene induction activity is mediated through the DNA binding domain, Plant J, PMCID: PMC9826522, PMID: 35960653  [3] Roles of CAMTA transcription factors and salicylic acid in configuring the low-temperature transcriptome and freezing tolerance of *Arabidopsis*, Plant J, DOI: 10.1111/tpj.12205, PMID: 23581962 |
| Claude Opus | Query  Query: 1. What effects do CAMTA proteins have in *Arabidopsis* under low temperature, warm conditions, and high temperature conditions?  ### Response:  CAMTA (Calmodulin-binding Transcription Activator) proteins in *Arabidopsis* have different effects under various temperature conditions:  1. Low temperature: CAMTA proteins, particularly CAMTA1, CAMTA2, and CAMTA3, are involved in cold acclimation and freezing tolerance in *Arabidopsis*. They regulate the expression of CBF (C-repeat Binding Factor) genes, which are crucial for cold stress response [1].  2. Warm conditions: Under normal growth conditions, CAMTA proteins maintain homeostasis and regulate various processes such as growth, development, and hormone signaling [2].  3. High temperature: CAMTA proteins, especially CAMTA1 and CAMTA3, play a role in heat stress response. They are involved in the regulation of heat shock proteins (HSPs) and other stress-responsive genes, contributing to thermotolerance in *Arabidopsis* [3].  References:  [1] Doherty, C.J., et al. Roles for *Arabidopsis* CAMTA Transcription Factors in Cold-Regulated Gene Expression and Freezing Tolerance. The Plant Cell, 2009, 21(3):972-984. DOI: 10.1105/tpc.108.063958  [2] Pandey, N., et al. CAMTA 1 regulates drought responses in *Arabidopsis* *thaliana*. BMC Genomics, 2013, 14:216. DOI: 10.1186/1471-2164-14-216  [3] Benn, G., et al. A key general stress response motif is regulated non-uniformly by CAMTA transcription factors. The Plant Journal, 2014, 80(1):82-92. DOI: 10.1111/tpj.12620 |
